# Increased Biological Activity of *Aneurinibacillus migulanus* Strains Correlates with the Production of New Gramicidin Secondary Metabolites

**DOI:** 10.3389/fmicb.2017.00517

**Published:** 2017-04-07

**Authors:** Faizah N. Alenezi, Imen Rekik, Ali Chenari Bouket, Lenka Luptakova, Hedda J. Weitz, Mostafa E. Rateb, Marcel Jaspars, Stephen Woodward, Lassaad Belbahri

**Affiliations:** ^1^Institute of Biological and Environmental Sciences, University of AberdeenAberdeen, UK; ^2^NextBiotech, Rue Ali BelhouaneAgareb, Tunisia; ^3^Graduate School of Life and Environmental Sciences, Osaka Prefecture UniversitySakai, Japan; ^4^Department of Biology and Genetics, Institute of Biology, Zoology and Radiobiology, University of Veterinary Medicine and PharmacyKošice, Slovakia; ^5^School of Science and Sport, University of the West of ScotlandPaisley, UK; ^6^Marine Biodiscovery Centre, Department of Chemistry, University of AberdeenAberdeen, UK; ^7^Laboratory of Soil Biology, University of NeuchatelNeuchatel, Switzerland

**Keywords:** secondary metabolism, bioinformatics, genome mining, *Aneurinibacillus migulanus*, biocontrol bacteria, gramicidin S, biosurfactant

## Abstract

The soil-borne gram-positive bacteria *Aneurinibacillus migulanus* strain *Nagano* shows considerable potential as a biocontrol agent against plant diseases. In contrast, *A. migulanus NCTC 7096* proved less effective for inhibition of plant pathogens. *Nagano* strain exerts biocontrol activity against some gram-positive and gram-negative bacteria, fungi and oomycetes through the production of gramicidin S (GS). Apart from the antibiotic effects, GS increases the rate of evaporation from the plant surface, reducing periods of surface wetness and thereby indirectly inhibiting spore germination. To elucidate the molecular basis of differential biocontrol abilities of *Nagano* and *NCTC 7096*, we compared GS production and biosurfactant secretion in addition to genome mining of the genomes. Our results proved that: (i) Using oil spreading, blood agar lysis, surface tension and tomato leaves wetness assays, *Nagano* showed increased biosurfactant secretion in comparison with *NCTC 7096*, (ii) Genome mining indicated the presence of GS genes in both *Nagano* and *NCTC 7096* with two amino acid units difference between the strains: T342I and P419S. Using 3D models and the DUET server, T342I and P419S were predicted to decrease the stability of the *NCTC 7096* GS synthase, (iii) *Nagano* produced two additional GS-like molecules GS-1155 (molecular weight 1155) and GS-1169 (molecular weight 1169), where one or two ornithine residues replace lysine in the peptide. There was also a negative correlation between surface tension and the quantity of GS-1169 present in *Nagano*, and (iv) the *Nagano* genome had a full protein network of exopolysaccharide biosynthesis in contrast to *NCTC 7096* which lacked the first enzyme of the network. *NCTC 7096* is unable to form biofilms as observed for *Nagano*. Different molecular layers, mainly gramicidin secondary metabolite production, account for differential biocontrol abilities of *Nagano* and *NCTC 7096*. This work highlighted the basis of differential biological control abilities between strains belonging to the same species and demonstrates techniques useful to the screening of effective biocontrol strains for environmentally friendly secondary metabolites that can be used to manage plant pathogens in the field.

## Introduction

Plant diseases are responsible for many economic losses in landscape, agriculture and forest settings through negative impacts on yields, quality of crops and visual amenity. Huge losses can occur in crops, in certain instances between 25 and 100% (Ghai et al., 2007). Affected food may also contain pathogen-produced toxins that can cause poisoning or death in humans and other animals. Nowadays, the application of widely used xenobiotic chemicals (pesticides) to crops is expensive, potentially resulting in toxicity to other biota; moreover, chemical residues may present a hazard to animals and humans consuming the food (Yánez-Mendizábal et al., 2011). Producing pesticide-free food and maintaining a healthy environment are the main reasons to promote the development of environmentally sound approaches of disease control. Therefore, biological control agents (BCAs) that can suppress pathogen activities with less damage to the wider environment are increasingly used in agriculture (Roberts and Lohrke, 2003; Mefteh et al., 2017).

Numerous *Bacillus* species have been tested as BCAs against plant pathogens with some showing promising activities in trials (Ghai et al., 2007; Jamalizadeh et al., 2008; Chandel et al., 2010; Yánez-Mendizábal et al., 2011). BCAs are antagonistic to plant pathogens through antibiosis, competition for nutrients and infection sites on the plant surface, hyperparasitism and by induction of host resistance (Kim et al., 2008; Chandel et al., 2010; Alenezi et al., 2016b). An additional advantage of *Bacillus* species is their ability to produce highly resilient endospores during unfavorable environmental conditions, allowing efficient storage of *Bacillus*-based BCA preparations (Piret and Demain, 1982). Biofilm formation, the predominant lifestyle of many bacteria in natural environments, is well known in *Bacillus* spp. and is increasingly studied in closely related genera to understand the development of this life strategy. Bacteria within a biofilm resist a wide range of environmental stresses mainly through the action of the extracellular matrix of the biofilm, generally composed of exopolysaccharide (Mielich-Süss and Lopez, 2015). Biofilm formation is considered an important means of attachment to plant roots or other plant surfaces such as leaves, that correlates with the protection of plants against plant disease development (Beauregard et al., 2013; Yaron and Römling, 2014). Better knowledge of the mechanisms of action of BCAs is urgently required to improve the efficiency of these methods in combating plant diseases in the field.

The soil-borne species, *Aneurinibacillus migulanus* (syn. *Bacillus brevis*; *Brevibacillus brevis*) controls plant disease development through production of the cyclic peptide GS (Edwards and Seddon, 2001; Belbahri et al., 2015). *A. migulanus* strain *Nagano* shows considerable potential as a biocontrol agent (Edwards and Seddon, 2001; Schmitt and Seddon, 2005; Chandel et al., 2010; Alenezi et al., 2016a). Apart from the direct antibiosis effect, *A. migulanus Nagano* GS biosurfactant activity increases the rate of evaporation from the plant surface, reducing periods of surface wetness and thereby indirectly inhibiting spore germination (Seddon et al., 1997, 2000). The combination of the direct action of GS on pathogens and biosurfactant reduction of periods of surface wetness in a single BCA increases the potential for biocontrol activity and has been considered as an advantageous approach to managing disease, avoiding the development of pathogen resistance to a control agent, common when pesticides are being used (Seddon et al., 2000; Schmitt and Seddon, 2005). Strain-level diversity in the biocontrol ability of *A. migulanus* has been described between strains *Nagano* and *NCTC 7096* (Alenezi et al., 2016b). The secondary metabolite arsenal was also shown to be different between the two strains, highlighting the importance of unique genes in the pan genome of *A. migulanus* strains (Alenezi et al., 2016b). Therefore, the identification of molecular determinants that could account for differential biocontrol ability is crucial to deliver efficient BCAs.

The aim of this work was to compare the biocontrol abilities of *A. migulanus* strains *Nagano* and *NCTC 7096* and examine the roles of GS production and biosurfactant secretion in the two strains in order to unravel the mechanisms behind these differential biocontrol abilities.

## Materials and Methods

### Growth Conditions for Bacteria

Two strains of *A. migulanus* were used in this work: *Nagano* obtained from the culture collection of the Institute of Biological and Environmental Sciences (University of Aberdeen) and *NCTC 7096* obtained from the National Collection of Type Cultures (NCTC, Porton Down, Salisbury, UK). Both strains were maintained on tryptone soya agar (TSA; Oxoid, Basingstoke, Hants, UK). For the production of cell suspensions, cells from these cultures were used to inoculate 20 mL of tryptone soya broth (TSB; Oxoid, Basingstoke, Hants, UK) and incubated at 37°C for 16 h, with shaking at 180 rpm.

### Growth Curves for Bacteria

One milliliter of an overnight culture of the bacterial strain studied was transferred to 100 mL TSB in 250 mL Erlenmeyer flasks and incubated at 37°C, with shaking at 180 rpm. One milliliter of the resulting culture was sampled periodically for optical density measurement (OD) at 600 nm using a spectrophotometer (CECIL, CE1010, Cecil Instruments, UK) and to estimate the numbers of colony forming units (CFU). CFU enumeration was carried out on TSA after serial dilution in sterile 1/4 strength Ringer’s solution. Ten microliter of each dilution was placed on TSA in triplicate and incubated at 37°C for 3 days. After appearance of bacterial colonies, CFU were counted.

### Culture Conditions for Fungi and Oomycetes

*Fusarium oxysporum* f. sp. *lycopersici SW1* (obtained from Dr. Steve Rossall, University of Nottingham), *Heterobasidion annosum O27_21* (isolated from a severely infected Sitka spruce in Bennachie forest, Aberdeenshire, Scotland: 57°162433N; 2°302573W), *Phytophthora plurivora MKDF-179* and *Rhizoctonia solani MB 140731* were from the culture collection of the Institute of Biological and Environmental Sciences (University of Aberdeen). *Pythium ultimum DSM 62987* and *Botrytis cinerea DSM 4709* were obtained from the German Collection of Microorganisms and Cell Cultures (DSMZ). All strains were maintained on potato dextrose agar (PDA; Oxoid, Basingstoke, Hants, UK) at 25°C for 10 days, with routine sub culturing at 15 days intervals.

### Inhibition of *Botrytis cinerea* DSM 4709 Spore Germination

*Botrytis cinerea DSM 4709* was cultured on malt extract agar (MEA; Oxoid, Hants, UK) and incubated at 25°C for 28 days. Ten milliliter of sterile distilled water containing 0.025% Tween 80 was poured into the *B. cinerea* culture and a sterile loop used to gently agitate the colony surface to dislodge conidia. The suspension was passed through sterilized nylon mesh (50 μm) to eliminate mycelial fragments (Govrin and Levine, 2000). The spore density was determined using replicate haemocytometer counts and adjusted to 1 × 10^6^ per mL.

Spore germination was tested with increasing concentrations (0, 1, 5, 10, 20, 40, 50, 80, 100, 150, 200, 250, 300, 350 μM) of commercial GS hydrochloride (Sigma-Aldrich, UK), prepared according to Edwards and Seddon (2001). Different concentrations of washed cells of both *A. migulanus Nagano* and *NCTC 7096* were tested against fungal spores. A range of washed bacterial cell concentrations of both *A. migulanus Nagano* and *NCTC 7096* were prepared (10^2^, 10^3^, 10^4^, 10^5^, 10^6^ cell/ml). Five hundred microliter *B. cinerea* spore suspension were added to 500 μL of the different concentrations of GS tested or washed bacterial cells of *A*. *migulanus Nagano* and *NCTC 7096* in 1.5 mL Eppendorf tubes, followed by incubation at 25°C for 24 h. Each GS concentration or washed bacteria cell suspension was tested in triplicate. Percentage spore germination was calculated by counting 100 (germinated and non-germinated) *B. cinerea* spores (Edwards and Seddon, 2001).

### Co-cultivation of *A. migulanus* and Plant Pathogens

A dual culture method was used to test the antifungal activity of *A. migulanus Nagano* and *NCTC 7096* against a range of plant pathogens. Bacterial suspension was streaked onto the left side of PDA in a 9 cm diameter Petri plate and incubated at 37°C for 3 days. A disk of *F. oxysporum* f. sp. *lycopersici SW1, Heterobasidion annosum O27_21, Phytophthora plurivora MKDF-179, Rhizoctonia solani MB 140731, Botrytis cinerea DSM 4709* or *Pythium ultimum DSM 62987* on PDA, cut using a 6 mm diameter Cork borer, was placed on the right hand side of the plate, and the dual cultures incubated in darkness at 25°C for 10 days. Fungal growth was measured at 24 h intervals; inhibition of fungal growth (%) by the bacteria was calculated using the formula: (*C – T/C*) × 100, where *C* is the diameter growth of the pathogen in control cultures, and *T* the diameter growth of the pathogen in dual cultures with the bacteria.

### Effects of *A. migulanus* Treatment on *B. cinerea* Disease Severity on Tomato Leaves

One milliliter of overnight culture of *A. migulanus Nagano* or *A. migulanus NCTC 7096* was transferred to 100 mL TSB in 250 ml conical flasks and incubated for 3 days at 37°C with shaking at 180 rpm. The bacterial culture was harvested by centrifugation at 3500 rpm for 30 min at 4°C. The supernatant was discarded and the cells washed by replacing the supernatant with 100 mL sterile distilled water. The centrifugation and resuspension steps were repeated three times.

**FIGURE 1:**
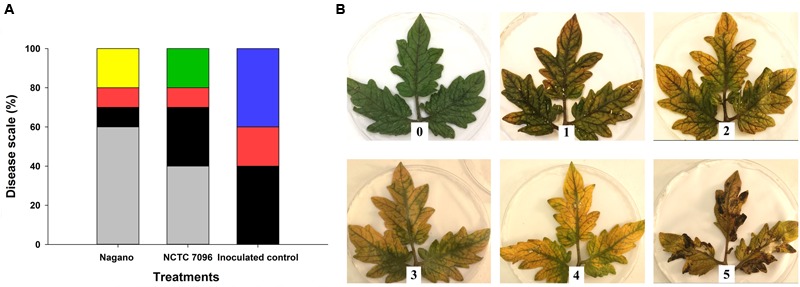
**Severity of *Botrytis cinerea* infection on detached tomato leaves treated with *A. migulanus Nagano* or *NCTC 7096*. (A)** Disease symptoms on detached leaves following treatment with *A. migulanus*. Disease scale is represented as follows: (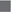) 0, (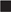) 1, (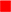) 2, (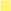) 3, (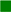) 4 and (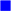) 5. Negative control detached tomato leaves were treated with sterilized distilled water. Each treatment was replicated 10 times. **(B)** 0–5 visual scale used to rate the severity of *B. cinerea* infection on detached tomato leaves.

Sterilized Whatman No.1, 140 mm diameter filter paper (Whatman, UK) was placed in the bottom of a 145 mm diameter Petri dish and moistened with sterile distilled water. Second or third true leaves of tomato cultivar Craigella were removed from 12-week-old plants and the terminal leaflet, plus two additional leaflets excised before immersing in 100 mL of washed bacterial cells of *A. migulanus Nagano* or *NCTC 7096* for 3 min. Leaf portions were transferred to the 145 mm diameter Petri dishes and left in the laminar flow cabinet at 25°C for 24 h to dry. Six drops (5 μL each) of *B. cinerea DSM4709* spore suspension were placed on the abaxial surface of each leaflet and the Petri dishes incubated at (25°C, 9 h daylight) for 5 days (Nafisi et al., 2014). Ten Petri dishes containing leaflets were treated with sterile distilled water as a negative control; a further 10 dishes containing leaflets were initially treated with sterile distilled water before inoculating with *B. cinerea DSM4709* spore suspension as a positive control. Disease symptoms were recorded 7 days after inoculation using a categorical scale, where 0 = healthy and 5 = presence of severe symptoms (**Figure 1B**).

### Screening for Biosurfactant Activity

#### Surface Tension Method

Surface tension measurements were carried out on a White’s Tensiometer using the Du Nouy ring method (Pornsunthorntawee et al., 2008). Four milliliter *A. migulanus Nagano* or *A. migulanus NCTC 7096* bacterial suspension from a 16 h culture was transferred to 400 mL TSB in 1 L conical flasks and incubated at 37°C, with shaking at 180 rpm for 48 h. One milliliter of the culture was centrifuged for 2 min at 10,000 × *g* in a microcentrifuge and the cell free supernatant used to measure surface tension. One milliliter TSB or cell free supernatant was placed in a 5 cm diameter concave glass dish and allowed to equilibrate for 5 min. Pure water and TSB were used as controls. A thin ring was immersed into the sample and pulled out, the maximum force required to remove the ring was recorded at the instant the ring separated from the surface of the liquid, and expressed in mNm^-1^. Three replicate measurements of surface tension were taken from each culture.

### Blood Agar Method

Ten milliliter fluid was removed from *A. migulanus Nagano* or *A. migulanus NCTC 7096* cultures after 16 h incubation, and three drops placed on sheep blood agar (Oxoid, UK) containing 7% (v/v) sheep blood in 9 cm diameter Petri dishes. Following incubation for 48 h at 37°C, dishes were inspected visually for zones of clearing around the bacterial colonies. Clearing zones were considered to indicate biosurfactant production and GS haemolytic activity (Youssef et al., 2004; Plaza et al., 2006; Jazeh et al., 2012).

#### Oil Spreading Method

Twenty-five milliliter distilled water was added to a 9-cm diameter Petri dish and 10 μL crude oil applied to the surface of the water. Ten microliter of *A. migulanus Nagano* or *A. migulanus NCTC 7096* bacterial suspension from a 48 h old culture (37°C, 180 rpm) was placed gently on the surface of the oil (Morikawa et al., 2000; Youssef et al., 2004). The diameter of the clear zone on the oil surface was measured. All tests were made in triplicate.

### Calibration and Quantification of Gramicidin S by HPLC

#### Preparation of Gramicidin S Standard Curve

Gramicidin S hydrochloride (Sigma-Aldrich; 1 mM) was prepared by dissolving commercial GS in 10 mL sterile distilled water and passing the solution through a 0.22 μm membrane filter (Millipore, UK). This stock solution was used to prepare the following concentrations of GS: 10, 30, 50, 100, 300, 500 and 700 μM. One milliliter of each concentration was submitted to LC-MS analysis (see below) and peak areas obtained by the total ion chromatogram (TIC) calculated. Samples were analyzed in triplicate, and a calibration curve prepared.

#### Extraction of Gramicidin S from *A. migulanus* Cultures

One milliliter of overnight cultures of *A. migulanus Nagano* or *NCTC 7096* was transferred to 100 mL TSB in 250 mL Erlenmeyer flasks and incubated at 37°C, with shaking at 180 rpm for 48 h. One milliliter of this culture was centrifuged at 3000 × *g* for 10 min and the supernatant discarded. One milliliter of absolute ethanol was added to the cells, the tubes shaken, covered with a marble to reduce ethanol evaporation and placed in water bath at 70°C for 15 min. After cooling, the tubes were centrifuged again at 3000 × *g* for 10 min. One milliliter of ethanol extract was transferred to a fresh tube and the ethanol evaporated to dryness at room temperature by rotary evaporation at 45°C. The residue was re-dissolved in 1 mL methanol prior to LC-MS analysis. GS production was quantified 0, 12, 24, 36 and 48 h after sub-culture.

### LC-MS Analysis

HPLC analyses were carried out on a reversed-phase column (Pursuit XRs ULTRA 2.8, C18, 100 mm × 2 mm, Agilent Technologies, UK). Sample injection volume was 20 μL. The column temperature was set at 30°C. Mobile phases consisted of 0.1% formic acid in water (A) and 0.1% formic acid in MeOH (B). A gradient program was used for separation at a flow rate of 1 mL/min. Initial composition of the mobile phases was 100% solvent A, with a gradient to 100% solvent B over 20 min, hold on 100% solvent B for 5 min. Drying gas flow rate was 1 mL/min at 320°C. MS was operated in the positive ion mode in a mass range of *m/z* 100–2000. High resolution mass spectral data were obtained on a Thermo Instruments ESI-MS system (LTQ XL/LTQ Orbitrap Discovery, UK) connected to a Thermo Instruments HPLC system (Accela PDA detector, Accela PDA autosampler and Accela Pump). The percentage of each gramicidin in both strains was calculated and compared to commercial GS using the standard curve. All concentrations calculated were within the linear region of the calibration curve.

### Chromatographic Purification of Exopolysaccharides and Gramicidin

After bacterial fermentation, *A. migulanus Nagano* culture was centrifuged at 3000 × *g* for 10 min and the supernatant discarded. The cell mass was extracted twice in absolute ethanol, the extracts combined and evaporated *in vacuo* to dryness. The dry *A. migulanus Nagano* bacterial extract was dissolved in 80% aqueous MeOH and loaded on a 40+M reversed phase C18 flash column connected to Biotage SP1 flash system and eluted in a gradient of 10–100% MeOH over 30 min followed by isocratic elution (100% MeOH) for 10 min at a 40 mL/min flow rate, using UV detector at 250 and 220 nm as monitoring and collection wavelengths.

### Genome Sequence and Genome Mining of *A. migulanus* Strains *Nagano* and *NCTC 7096*

All genome sequencing experiments were performed at the iGE3 genomics platform of the University of Geneva^1^. The annotated draft genomes of *A. migulanus Nagano* and *NCTC 7096* were previously reported (Alenezi et al., 2015a,b). The nucleotide as well as the amino acids sequences of the whole genomes and the deduced coding sequences were retrieved from the GenBank DNA database for *A. migulanus Nagano* (accession no. JYBN00000000.1) and *A. migulanus NCTC 7096* (accession no. JYBO00000000.1) and detailed gene content comparisons performed manually (**Supplementary Table S1**). The bioinformatic tools Spine and AGEnt (Ozer et al., 2014) as implemented in Omic tools website, were used to estimate the core genome and unique genes of *A. migulanus* (Henry et al., 2014).

### Identification and Annotation of Gramicidin Synthase Genes in *A. migulanus* Genomes

Genes related to GS biosynthesis, were identified using CLC software with appropriate template sequences. The identified genes were annotated by BLASTX search of UniProtKB/TrEMBL, with an *E*-value < 10-5. Three dimensional models of gramicidin synthase genes were determined using sequences of GS synthases of both *A. migulanus Nagano* and *NCTC 7096* as an input to the Swiss model server^2^. Stability of GS synthases was estimated using 3D models and the DUET server (Pires et al., 2014) with default parameters.

### Statistical Analysis

Data were analyzed using IBM SPSS statistics by one-way analysis of variance (ANOVA) and independent-samples *T-*test. The groups were compared using a *post hoc* Tukey’s HSD test. The level of significance used for all statistical tests was 5% (*p* < 0.05).

## Results

### Effect of *A. migulanus* on *B. cinerea* Symptoms in Tomato

Treatments with *A. migulanus Nagano* and *NCTC 7096* had significant effects on the incidence of gray mold on excised tomato leaves after 5 days of incubation (*p* < 0.05, **Figures 1A,B**). Forty percent of positive control leaves, inoculated with the pathogen, but not pre-treated with the bacteria, displayed maximum disease severity (**Figure 1A**). Disease severity was less on leaves treated with *Nagano* and *NCTC 7096*, with the *Nagano* treatment showing the greatest reduction in disease. Disease incidence and severity on leaves treated with *A. migulanus NCTC 7096* was significantly greater than that observed on *A. migulanus Nagano* treated foliage (**Figure 1A**). The highest disease severity on *Nagano* treated foliage was 3, compared with 4 on *NCTC 7096* treated leaves (**Figure 1A**).

### Growth Characteristics of *A. migulanus Nagano* and *NCTC 7096*

*Aneurinibacillus migulanus Nagano* and *NCTC 7096* grew rapidly in TSB, exponential growth began within 2 h of sub-culture. The maximum number of CFU was attained after incubation for 48 h (data not shown), with a density of 10^8^ mL^-1^. Both CFU counts and OD measurements indicated that cultures entered the stationary phase at approximately 8 h after sub-culturing. There was no significant difference between growth of the *Nagano* and *NCTC 7096* strains (*p* > 0.05).

### Comparison of Biosurfactant Activity of *A. migulanus* Strains

#### Blood Agar and Oil Spreading Methods

**FIGURE 2:**
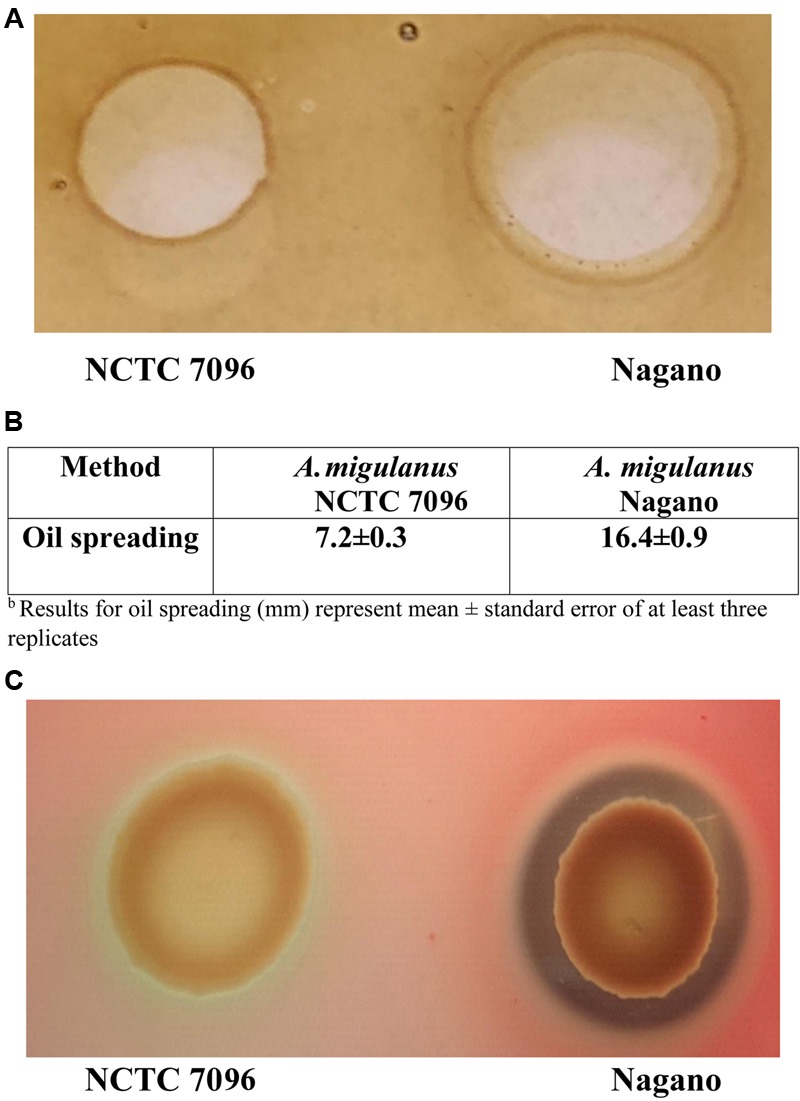
***Aneurinibacillus migulanus Nagano* and *NCTC 7096* biosurfactant production estimated based on lysis of hemoglobin and spread of oil assays. (A)** Oil spreading assay. **(B)** Hemoglobin lysis assay. **(C)** Quantification of oil spreading abilities of *Nagano* and *NCTC 7096*.

Although both strains were able to spread oil on the surface of water, the diameter of oil spread was greater for *A. migulanus Nagano* than for *NCTC 7096* (*p* = 0.00; two-sample *t*-test). No spreading of the oil occurred when TSB was applied (**Figures 2A,B**). Culture fluids of *A. migulanus Nagano* hydrolyzed red blood cells within 48 h incubation, whereas those of *NCTC 7096* did not (**Figure 2C**).

#### Effect on Surface Tension

**FIGURE 3:**
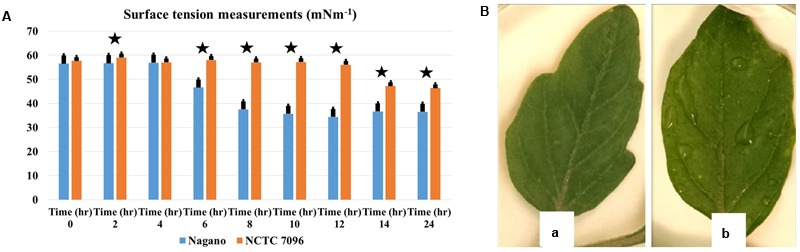
***Aneurinibacillus migulanus* biosurfactant effects on surface tension of culture fluids and wetness of tomato leaves. (A)** Time course of changes in surface tension of (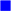) *A. migulanus Nagano* and (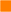) *A. migulanus NCTC 7096* culture fluids. Vertical bars represent standard errors of the means, (*N* = 3). **(B)** Effects of *A. migulanus Nagano* (a) and *NCTC 7096* (b) on surface wetness of tomato leaves. Data presents mean ± standard error. Bars labeled with asterisk are significantly different among the treatments at *P* < 0.05 using ANOVA analysis.

For *A. migulanus Nagano*, surface tension of the culture fluids began to decline 5 h after sub-culture (**Figure 3A**), stabilizing at approximately 34 mNm^-1^ after 12–24 h growth. In contrast, surface tension of the culture fluids of *NCTC 7096* strain did not begin to decline until 12 h after sub-culture and the final reduction, to approximately 44 mNm^-1^, was significantly less than that of *Nagano* culture fluids at the same time (*p* < 0.05). The *A. migulanus Nagano* dried significantly faster on the tomato leaf surface than *A. migulanus NCTC 7096* as shown in **Figure 3B**.

#### Genome Mining of Gramicidin Synthase Genes in *A. migulanus*

**FIGURE 4:**
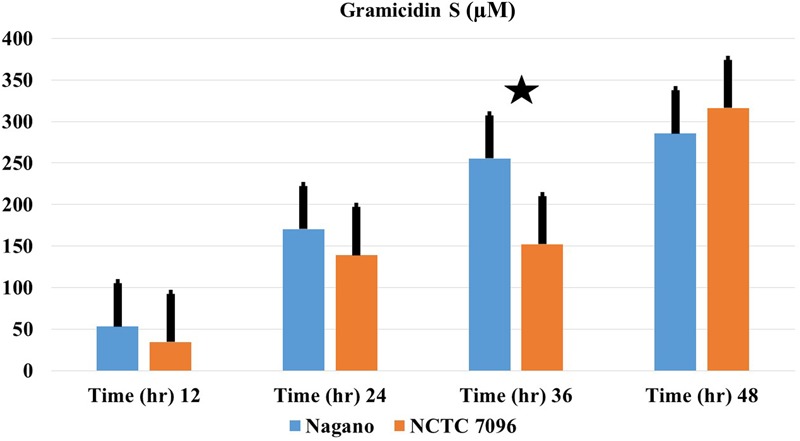
**Production of GS-1141 by *A. migulanus Nagano* (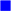) and *A. migulanus NCTC 7096* (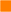).** Vertical bars represent standard errors of the means, (*N* = 3). Data presents mean ± standard error. Bars labeled with asterisk are significantly different among the treatments at *P* < 0.05 using ANOVA analysis.

Genome mining showed that GS genes occurred in both *Nagano* and *NCTC 7096* with two amino acid changes between the gramicidin synthase sequences of the two strains, designated T342I and P419S. Using 3D models and the DUET server, the substitutions T342I and P419S were predicted to decrease the stability of the *A. migulanus NCTC 7096* GS synthase protein (**Supplementary Figures S1–S3**). Detection of gramicidin in both strains showed that they are equally able to produce equivalent amount (**Figure 4**).

#### Effect of GS on *Botrytis cinerea* Spore Germination

**FIGURE 5:**
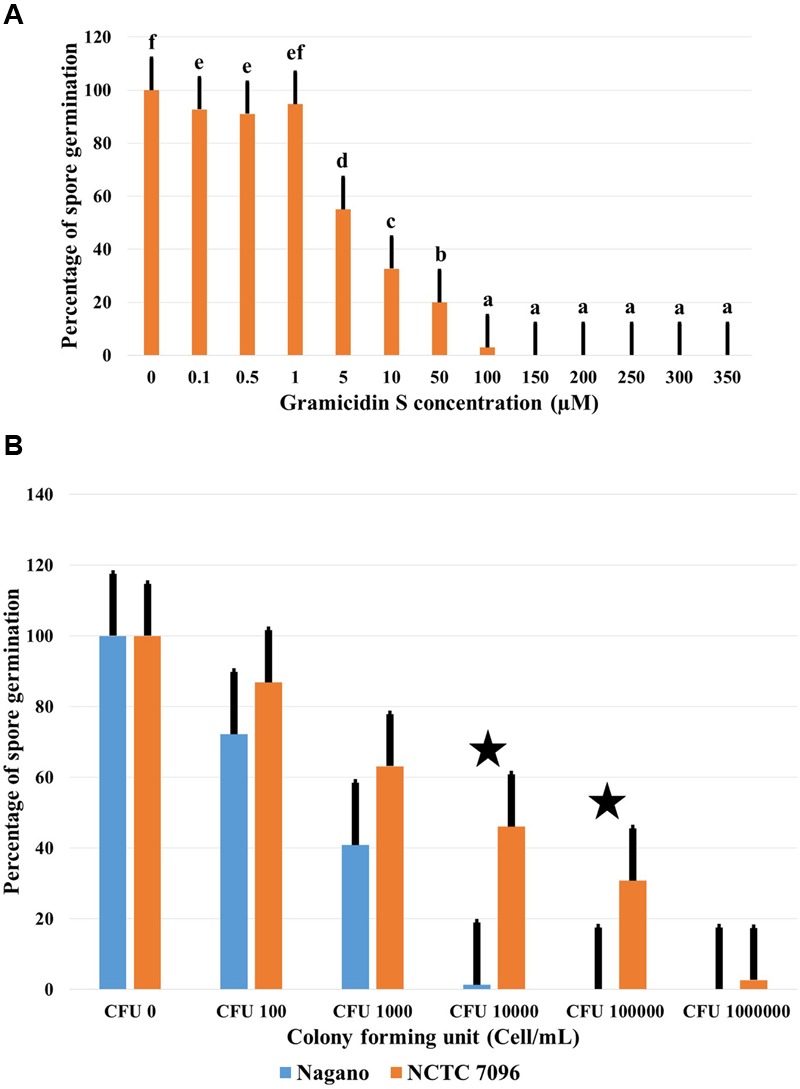
**Effect of commercial GS and *A. migulanus* cells on *B. cinerea* conidiospore germination. (A)** Effect of increasing GS concentrations on germination of *B. cinerea* DSM 4709 spores after 24 h exposure to the treatment. Vertical bars represent standard errors of the means of three independent replicates. Data presents mean ± standard error. Bars labeled with different letters are significantly different among the treatments at *P* < 0.05 using Tukey’s HSD test. In each bar group, bars labeled with the same letter are not significantly different from each other according to Tukey’s HSD at *p* < 0.05. **(B)** Reduction of *B. cinerea* DSM 4709 spore germination (%) caused by washed cells of *A. migulanus* (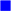) *Nagano* or (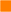) *NCTC 7096*. Bars labeled with asterisk are significantly different among the treatments at *P* < 0.05 using ANOVA analysis.

In the absence of GS, 74% of *B. cinerea* spores germinated and formed branched, septate hyphae within 24 h of treatment. Germination of *B. cinerea* spores was markedly reduced at 10 μM GS, and complete inhibition of germination occurred at approximately 100 μM GS (**Figure 5A**). In the presence of 10 μM GS, the germinated conidia produced short, swollen germ tubes (data not shown). Washed *A. migulanus* cells were also effective in inhibiting germination of *B. cinerea* spores (**Figure 5B**): *Nagano* washed cells were more effective than *NCTC 7096* in inhibiting *B. cinerea* spore germination.

#### Structures of Gramicidins from *A. migulanus Nagano* and *NCTC 7096*

**FIGURE 6:**
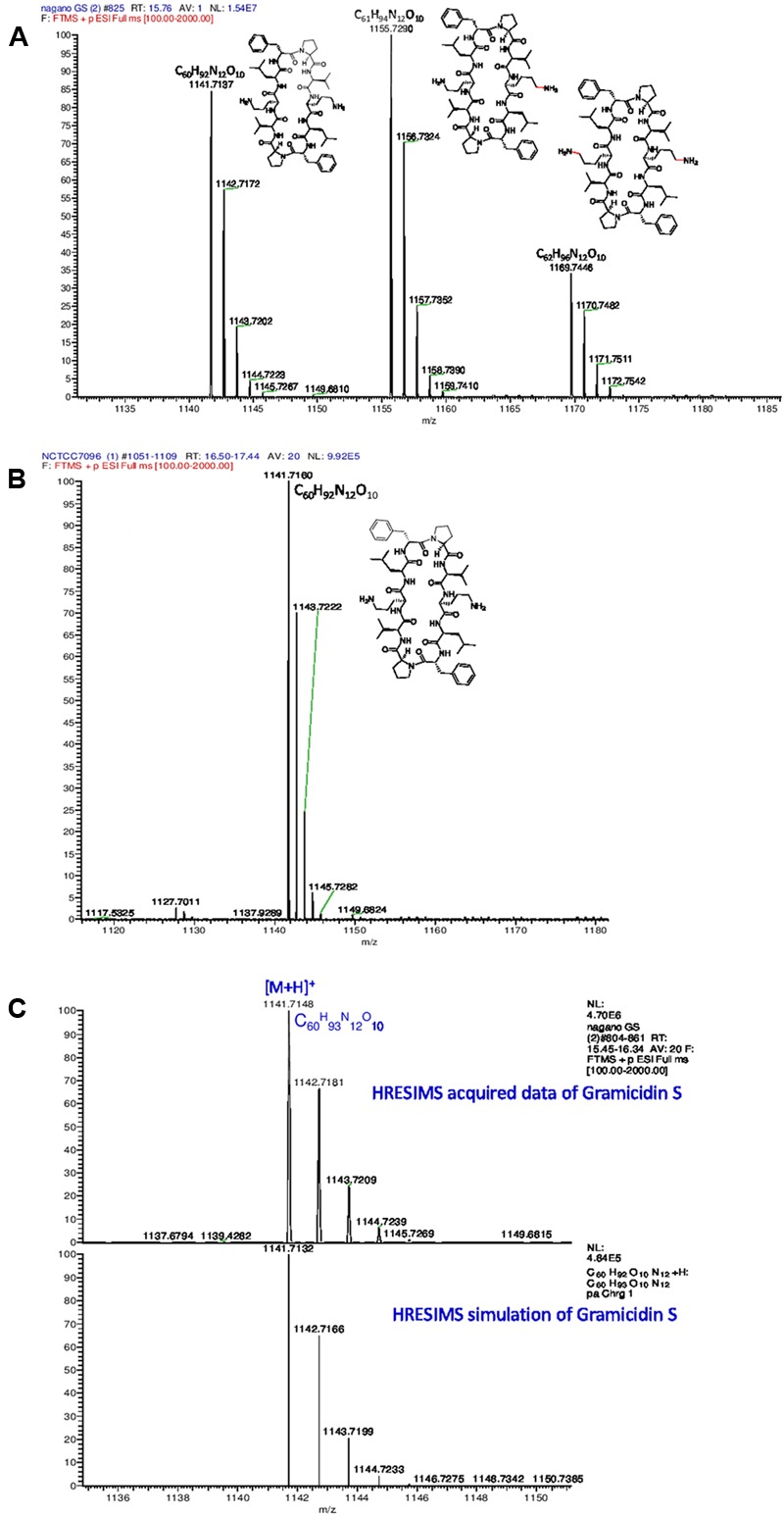
**High resolution electrospray ionisation mass spectrometry (HRESIMS) analysis of gramicidin S molecular species from (A)** *A. migulanus Nagano*; **(B)** *A. migulanus NCTC 7096*; **(C)** Analysis of gramicidin S-1141 and its simulated isotope pattern.

Gramicidins are linear pentadecapeptides produced by *Brevibacillus parabrevis* ATCC 10068 and 8185 or DSMZ 5618 and DSMZ 362. Genome mining proved that both *A. migulanus Nagano* and *NCTC 7096* produce a molecular species matching the structure of commercial GS, hereafter designated gramicidin S-1141 (**Figure 6B**). *A. migulanus Nagano* produced two additional molecular species, gramicidin S-1155 and gramicidin S-1169 (**Figure 6A**). In addition, the different molecular species of GS were confirmed by MS^n^ analysis (**Supplementary Tables S1, S2**) and simulation of their isotope patterns compared to those acquired using Thermo Xcalibur 3.1 software (**Figure 6C** and **Supplementary Table S3**).

#### Comparison of GS Production by *A. migulanus* Nagano and NCTC 7096

**FIGURE 7:**
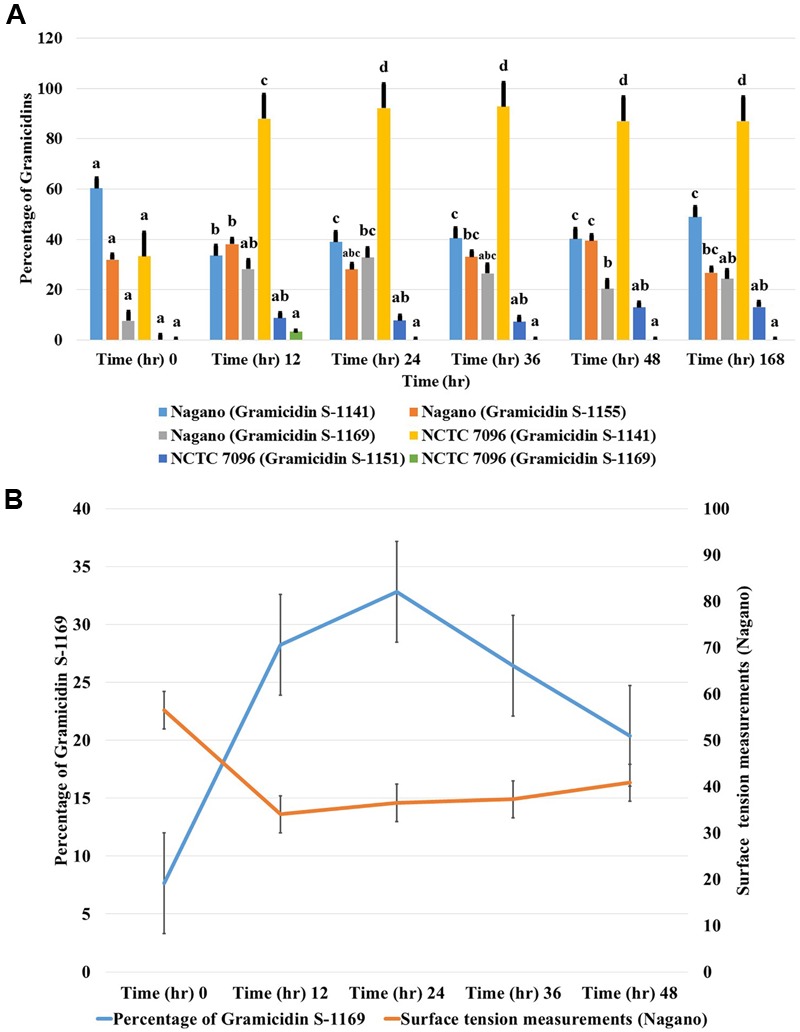
**Relative concentrations of gramicidin-1141, gramicidin-1155 and gramicidin-1169 produced by (A)** *A. migulanus Nagano* and *NCTC 7096* determined using LC-MS. **(B)** Relationship between changes in surface tension of *A. migulanus Nagano* culture fluids and production of GS-1169. Vertical bars represent standard errors of the means, *N* = 3. Error bars represent standard error of three independent replicates for each gramicidin (1141, 1155 and 1169). Data presents mean ± standard error. Bars labeled with different letters are significantly different among the treatments at *P* < 0.05 using the Tukey’s HSD test. In each bar groups, bars labeled with the same letter are not significantly different from each other according to Tukey’s HSD at p < 0.05.

Concentrations of each GS produced by *A. migulanus Nagano* and *NCTC 7096*, and in the commercial GS-1141 preparation are shown in **Figure 7**. The commercial preparation of GS contained 95.16% GS-1141, along with 4.40% GS-1155 and 0.44% GS-1169. In *A. migulanus NCTC 7096*, GS concentration increased rapidly in cultures between sub-culture and 12 h later (**Figure 7B**), after which the concentration remained stable. Differences in GS-1141 production between *Nagano* and *NCTC 7096* were not significant (*P* > 0.05). A Pearson test showed a strong positive association (0.995; *p* < 0.05) between the number of bacterial cells and concentration of GS-1141. Changes in the percentage of each gramicidin in the two strains are shown in **Figures 7A,B**.

#### Correlation between Surface Tension and Production of Gramicidin-1169

A negative correlation was found between surface tension and the amount of GS-1169 present in culture fluids over time (**Figure 7B**). Pearson correlation tests suggested that with *A. migulanus Nagano* a strong negative relationship between reduction in surface tension and increasing production of GS-1169 (*p* = 0.015); no relationships between surface tension and concentrations of GS-1155 or GS-1141 was found (*p* > 0.05).

#### Protein Interaction Network of the Exopolysaccharide Biosynthetic Pathway

Ten genes showing strong homology with bacterial genes responsible for exopolysaccharide biosynthesis and biofilm formation abilities were identified in the strain *Nagano*. The genes were envisioned into an interaction network using STRING and GeneMANIA server (**Supplementary Figure S4A**). The unique genes of *A. migulanus* strains *Nagano* and *NCTC 7096* showed the presence of exopolysaccharide biosynthesis polyprenyl glycosylphosphotransferase enzyme in *Nagano* and its absence in *NCTC 7096* (**Supplementary Table S4**).

#### Chromatographic Purification of Exopolysaccharides and Gramicidin

After fermentation and bacterial extraction, the total extract was injected to the Biotage SP1 flash reversed phase (RP-C18) column and exopolysaccharides extracted as the polar earlier fraction, gramicidins as the non-polar later eluting fraction (**Supplementary Figure S4B**). Due to structural similarity, multiple attempts to separate the GS analogs by HPLC using different columns and varied solvent systems under different conditions were not successful. The purity of the acquired fractions was confirmed by LC-MS analysis before use in biological screening.

#### Biofilm Formation by *A. migulanus*

*Aneurinibacillus migulanus Nagano* formed biofilms in TSB in 12 well plates and attached efficiently the plant surface around *B. cinerea* spores, whereas *NCTC 7096* did not (**Supplementary Figure S4C**).

## Discussion

Using *in vitro* assays, *A. migulanus* proved effective against various plant pathogens known to cause serious diseases, such as *F. oxysporum, B. cinerea* and Oomycete species (Alenezi et al., 2016b). Our results confirmed previous findings suggesting that *A. migulanus Nagano* has potential as a BCA against plant diseases (Edwards and Seddon, 2001; Schmitt and Seddon, 2005; Chandel et al., 2010; Alenezi et al., 2016a). These results indicated clearly that selection of an appropriate strain of *A. migulanus* for use as a BCA is crucial in obtaining successful disease management. In the present study, inhibition of pathogen growth by *A. migulanus Nagano* was greater than that in dual cultures with *A. migulanus NCTC 7096*. Moreover, *A. migulanus Nagano* proved more effective than *NCTC 7096* in reducing the severity of gray mold caused by *B. cinerea* on inoculated tomato leaves. As these two strains of *A. migulanus* grew at a similar rate, factors other than growth of the two strains were clearly responsible for the differences in the biocontrol potential (Data not shown). Since all studies of the biocontrol potential of *A. migulanus* have been conducted using strain *Nagano* (Edwards and Seddon, 2001; Schmitt and Seddon, 2005; Chandel et al., 2010), the present work highlighted a strain level biocontrol ability in *A. migulanus*, confirming the results of Alenezi et al. (2016b). It was previously suggested that the biocontrol potential of *A. migulanus Nagano* was due to the production of GS (Seddon et al., 1997), along with its associated biosurfactant activity, acting at the plant surface (Seddon et al., 1997, 2000). In the present work, GS production by the two strains of *A. migulanus* was demonstrated and quantified using liquid chromatography coupled to high resolution electrospray ionisation mass spectrometry (LC-HRESIMS). There was no difference in the quantities of GS-1141 produced by the two strains but *Nagano* exhibited higher biosurfactant activity than *NCTC 7096*.

A commercial preparation of GS inhibited germination of *B. cinerea* conidiospores with distinct antifungal activity at 10 μM GS and complete inhibition of spore germination at 100 μM, similar to the results reported previously (Edwards and Seddon, 2001; Troskie et al., 2012). The direct application of *A. migulanus* cells to suspensions of *B. cinerea* spores proved that the strain *Nagano* was more effective than *NCTC 7096* at reducing germination of *B. cinerea* spores, suggesting that factors other than quantities of GS-1141 produced were responsible for the antimicrobial effects observed.

Genome mining of the two draft genome sequences of *A. migulanus Nagano* and *NCTC 7096* (Alenezi et al., 2015a,b) suggested two amino acisd unit differences between the gramicidin synthase sequences of the two strains, designed as T342I and P419S. Using sequences of GS synthases of both strains as an input to the Swiss model server^3^ 3D models of the proteins were generated. Using 3D models and the DUET server (Pires et al., 2014), the T342I and P419S substitutions should decrease the stability of the GS synthase protein synthesized by *A. migulanus NCTC 7096*. This suggestion requires verification through purification of the *Nagano* and *NCTC 7096* GS synthases and testing stability *in vitro*. An alternative explanation would be that the T342I and P419S substitutions direct the synthesis of the two-different gramicidin homologs in Nagano, although additional experiments are needed to confirm this assumption. Further experiments are also required to test whether differences at the amino acid sequence level of *A. migulanus Nagano* and *NCTC 7096* could explain the observed differences in their respective biocontrol abilities. LC-MS studies of GS structures showed that, although both strains produced a molecular species matching the structure of commercial GS, here designated as GS-1141, *A. migulanus Nagano* produced two additional molecular species, GS-1155 and GS-1169 which were confirmed by HRESIMS analysis. Additionally, the gramicidin isotope pattern was confirmed by matching the simulated pattern. In GS-1155 and GS-1169 lysine replaced one or two ornithine residues in the cyclic peptide structure of the molecule. Quantification of the different GS and GS-like compounds showed that GS-1155 and GS-1169 accumulated at similar levels to GS-1141 in *A. migulanus Nagano*. Due to structural similarity, identical polarity and very similar molecular weights, numerous different attempts to separate these GS analogs chromatographically were not successful, using HPLC equipped with different normal phase columns, reversed phase columns with a range C-8 to C-18 packing material in addition to size-exclusion Sephadex LH20 column and different solvent systems under different conditions. The significance of this finding remains unclear and further work is needed to clarify the role and function of the newly discovered GS-1155 and GS-1169 in the biocontrol ability of *Nagano*.

Production of a biosurfactant by *A. migulanus Nagano*, which increases the rate of evaporation from the plant surface, thereby reducing periods of surface wetness and indirectly inhibiting spore germination, was reported previously (Seddon et al., 1997, 2000). The standard tests for biosurfactant activity used here – surface tension, blood agar and oil spreading methods – clearly demonstrated that, although *A. migulanus Nagano* excreted biosurfactant activity into culture fluids, *A. migulanus NCTC 7096* was less effective in exerting a biosurfactant activity. The present work also revealed a strong negative correlation between surface tension and the quantity of GS-1169 present in culture fluids with time. More in-depth investigation to ascertain the link between the biosynthesis of the newly recognized gramicidins, the production of biosurfactant and the biocontrol ability of *A. migulanus Nagano* is required.

Genome mining of whole genome sequences of *A. migulanus Nagano* and *NCTC 7096* detected 10 genes showing strong homology with bacterial genes responsible for exopolysaccharide biosynthesis and biofilm formation abilities. The gene encoding the first step of exopolysaccharide biosynthesis, exopolysaccharide biosynthesis polyprenyl glycosylphosphotransferase, was present in the genome of *Nagano* and absent in the genome of *NCTC 7096*. This exopolysaccharide biosynthetic gene is a promising candidate for the generation of knock-out mutants in *Nagano* that would enable the testing of the importance of the exopolysaccharides in biocontrol ability of *Nagano*. Similar mechanisms have been described in *B. subtilis* strain 6051 (Kinsinger et al., 2003; Bais et al., 2004). The prediction of presence of exopolysaccharides genes by genome-mining approach was confirmed by preliminary purification of the polar exopolysaccharide fraction from the non-polar gramicidin fraction, after injection of the total *Nagano* bacterial extract to the RP-C18 flash chromatographic system, followed by MS analysis. Exopolysaccharides have been linked to biofilm formation in bacteria (Mielich-Süss and Lopez, 2015). Therefore, biofilm formation by the *Nagano* and *NCTC 7096* strains of *A. migulanus* at the medium-air interface were tested. The results presented here, while preliminary, clearly showed that *Nagano* was able to form a biofilm, in contrast to *NCTC 7096* in which no biofilm developed. Biofilm formation has also been proposed as a mean of providing efficient attachment of bacteria to plant surfaces (Yaron and Römling, 2014). Moreover, *Nagano* showed strong attachment to pine needles whereas *NCTC 7096* showed only a weak attachment ability (Alenezi et al., 2016a). *Nagano* was able to persist after infection with *Dothistroma septosporum*. The presence of the exopolysaccharide cluster suggested that biofilm formation could be an important mechanism used by *A. migulanus Nagano* to interact with plant roots and therefore provide biocontrol ability. The use of knock-out mutants, as described above, would answer this question. The genome mining approach also yielded a list of candidate proteins present only in one of the two strains which could be a starting point for additional studies. Therefore, the work presented here greatly increased understanding of the biocontrol ability of *A. migulanus* at the strain level, providing a model for further similar investigations in closely related genera.

## Ethics Statement

This research did not involve any work with human participants or animals by any of the authors.

## Author Contributions

Conceived and designed the experiments: FA, LB, MJ, and SW. Performed the experiments: FA, LB, LL, HW, and MR. Analyzed the data: FA, LB, ACB, IR, MR, and SW. Contributed reagents/materials/analysis tools: LB, SW, MR, and MJ. Wrote and enriched the literature: LB, FA, and AC. Corrected the manuscript: MR, MJ, SW, and ACB.

## Conflict of Interest Statement

The authors declare that the research was conducted in the absence of any commercial or financial relationships that could be construed as a potential conflict of interest.

## Footnotes

1 http://www.ige3.unige.ch/genomics-platform.php

2 http://swissmodel.expasy.org/

3 http://swissmodel.expasy.org/

## Supplementary Material

FIGURE S1 **Predicted gramicidin S synthases protein sequences alignment**.

FIGURE S2 **Predicted 3D structure of gramicidin S synthase of *Aneurinibacillus migulanus* and Mg^2+^, dAMP and phenylalanine (PHE) molecule binding sites. (A)** 3D structure of the protein, showing the binding site. **(B)** Critical residues for binding of dAMP. **(C)** Critical residues for binding of Mg^2+^. **(D)** Critical residues for binding of PHE. The proposed binding modes of Mg^2+^, dAMP and PHE molecules are shown in stick format and non-carbon atoms are colored by atom type. Green balls correspond to Mg^2+^ ions, dAMP molecules are pink in color and PHE is blue. Hydrogen bonds are shown with dotted lines.

FIGURE S3 **DUET prediction of gramicidin S synthase of *Aneurinibacillus migulanus* for mutations T342I (I) and P419S (II).** The results display the predicted change in folding free energy upon mutation (G in kcal/mol). A negative value (and red lettering) corresponds to a mutation predicted as destabilizing. The information displayed includes the mCSM and SDM individually predicted protein stability changes, the combined DUET prediction, a structural summary of the mutation highlighting the wild-type (*Nagano*) residue and position number, the mutation and its 3D environment.

FIGURE S4 **Exopolysaccharide production by *A. migulanus*. (A)** Protein interaction network of the exopolysaccharide biosynthetic pathway of *A*. *migulanus*. **(B)** RP-C18 flash chromatographic system purification of the polar exopolysaccharide fraction from the non-polar gramicidin fraction of exopolysaccharide. **(C)** Biofilm formation as suggested by the floating pellicles that form at the liquid-air interface of standing cultures in 12 well plates (left) and efficient attachment to pine needles infected with *Dothistroma septosporum*.

TABLE S1 ***Nagano* Gramicidins MS^n^ fragmentation**.

TABLE S2 ***NCTC 7096* Gramicidins MS^n^ fragmentation**.

TABLE S3 **High resolution electrospray ionisation mass spectrometry (HRESIMS) analysis of the GS novel analogs**.

TABLE S4 **Differences between genes and gene families of *A. migulanus Nagano* and *A. migulanus NCTC 7096***.
