# Supplementary material for: Increased Biological Activity of Aneurinibacillus migulanus Strains Correlates with the Production of New Gramicidin Secondary Metabolites
Source: Front Microbiol. 2017 Apr 7;8:517. doi: 10.3389/fmicb.2017.00517 (PMC5383652; doi:10.3389/fmicb.2017.00517)
Supplement: FIGURE S1 — Predicted gramicidin S synthases protein sequences alignment. [file Data_Sheet_1.PDF]

Figure S1

```
                                                301        .         .         .         .         :         .         .         .         .         4 400
  1 A. migulanus NCTC 7096   100.0%     AGSATSPSLVNKWKEKVTYINAYGPTETTICATTWVATKETIGHSVPIGAPIQNTQIYIVDENLQLKSVGEAGELCIGGEGLARGYWKRPELTSQKFVDN
  2 A. migulanus Nagano       99.7%     AGSATSPSLVNKWKEKVTYINAYGPTETTICATTWVATKETTGHSVPIGAPIQNTQIYIVDENLQLKSVGEAGELCIGGEGLARGYWKRPELTSQKFVDN
    consensus/100%                      AGSATSPSLVNKWKEKVTYINAYGPTETTICATTWVATKEThGHSVPIGAPIQNTQIYIVDENLQLKSVGEAGELCIGGEGLARGYWKRPELTSQKFVDN

                                                401        .         .         .         .         :         .         .         .         .         5 500
  1 A. migulanus NCTC 7096   100.0%     PFVPGEKLYKTGDQARWLSDGNIEYLGRIDNQVKIRGHRVELEEVESILLKHMYISETAVSVHKDHQEQPYLCAYFVSEKHIPLEQLRQFSSEELPTYMI
  2 A. migulanus Nagano       99.7%     PFVPGEKLYKTGDQARWLPDGNIEYLGRIDNQVKIRGHRVELEEVESILLKHMYISETAVSVHKDHQEQPYLCAYFVSEKHIPLEQLRQFSSEELPTYMI
    consensus/100%                      PFVPGEKLYKTGDQARWLsDGNIEYLGRIDNQVKIRGHRVELEEVESILLKHMYISETAVSVHKDHQEQPYLCAYFVSEKHIPLEQLRQFSSEELPTYMI

                                                501        .         .         .         .         :         .         .         .         .         6 600
  1 A. migulanus NCTC 7096   100.0%     PSYFIQLDKMPLTSNGKIDRKQLPEPDLTFGMRVDYEAPRNEIEETLVTIWQDVLGIEKIGIKDNFYALGGDSIKAIQVAARLHSYQLKLETKDLLKYPT
  2 A. migulanus Nagano       99.7%     PSYFIQLDKMPLTSNGKIDRKQLPEPDLTFGMRVDYEAPRNEIEETLVTIWQDVLGIEKIGIKDNFYALGGDSIKAIQVAARLHSYQLKLETKDLLKYPT
    consensus/100%                      PSYFIQLDKMPLTSNGKIDRKQLPEPDLTFGMRVDYEAPRNEIEETLVTIWQDVLGIEKIGIKDNFYALGGDSIKAIQVAARLHSYQLKLETKDLLKYPT

                                                601        .         .         .         .         :         .         .         .         .         7 700
  1 A. migulanus NCTC 7096   100.0%     IDQLVHYIKDSKRRSEQGIVEGEIGLTPIQHWFFEQQFTNMHHWNQSYMLYRPNGFDKEILLRVFNKIVEHHDALRMIYKHHNGKIVQINRGLEGTLFDF
  2 A. migulanus Nagano       99.7%     IDQLVHYIKDSKRRSEQGIVEGEIGLTPIQHWFFEQQFTNMHHWNQSYMLYRPNGFDKEILLRVFNKIVEHHDALRMIYKHHNGKIVQINRGLEGTLFDF
    consensus/100%                      IDQLVHYIKDSKRRSEQGIVEGEIGLTPIQHWFFEQQFTNMHHWNQSYMLYRPNGFDKEILLRVFNKIVEHHDALRMIYKHHNGKIVQINRGLEGTLFDF
```

Figure S2:

A

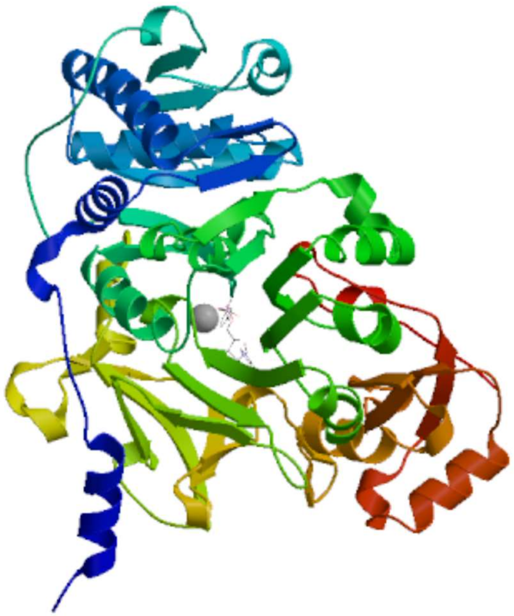

B

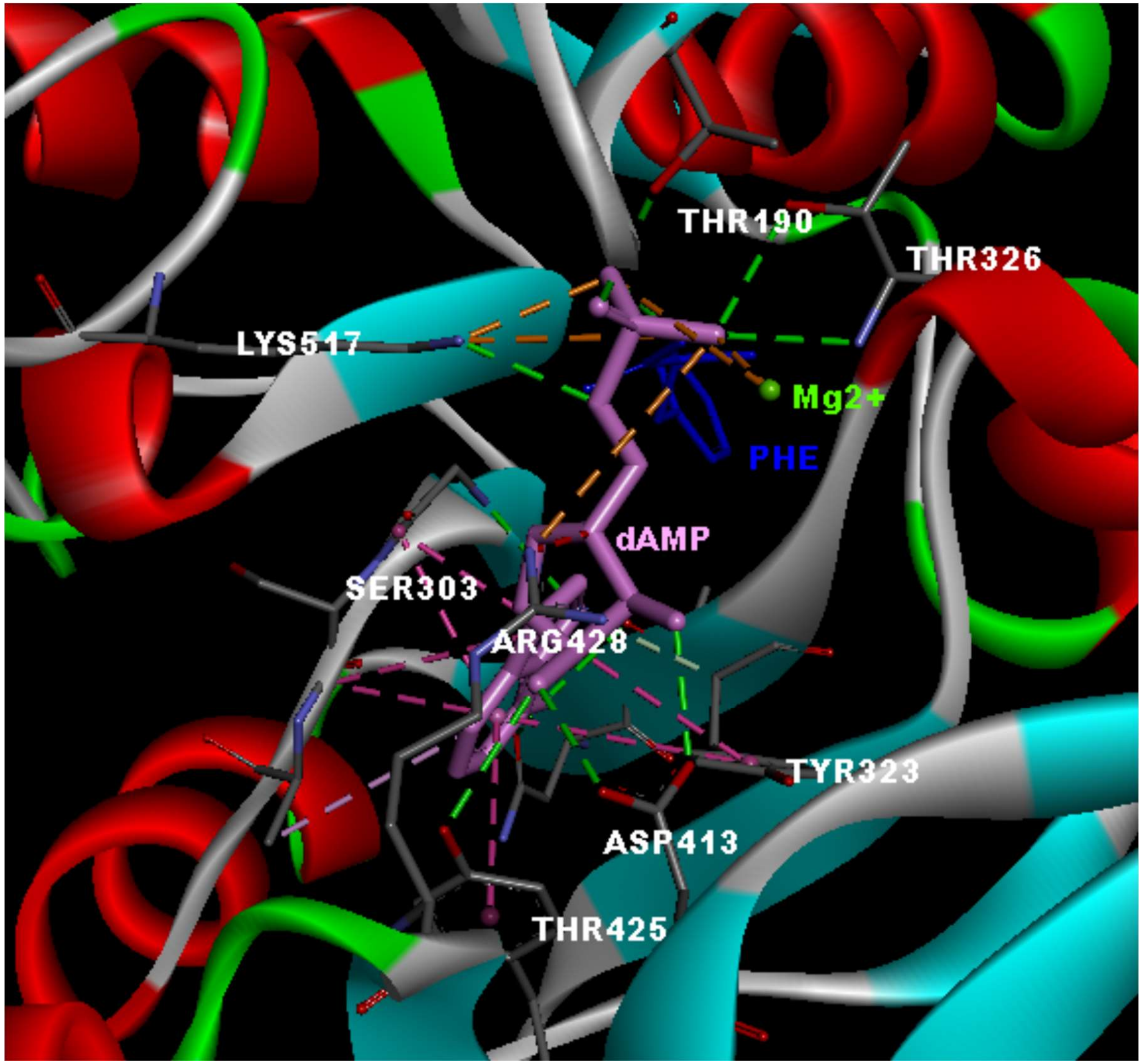
THR190
THR326
LYS517
Mg2+
PHE
dAMP
SER303
ARG428
TYR323
ASP413
THR425

C

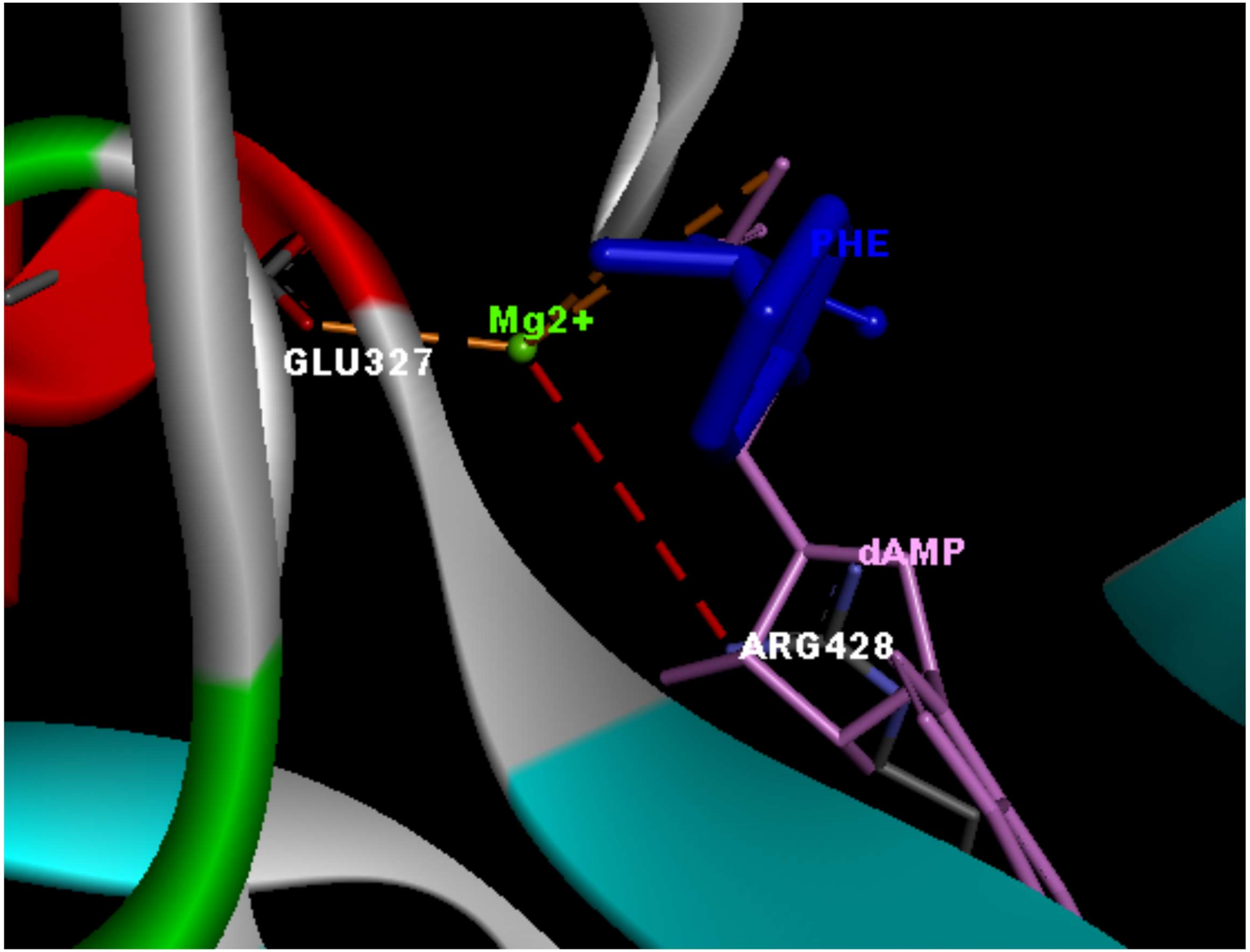

D

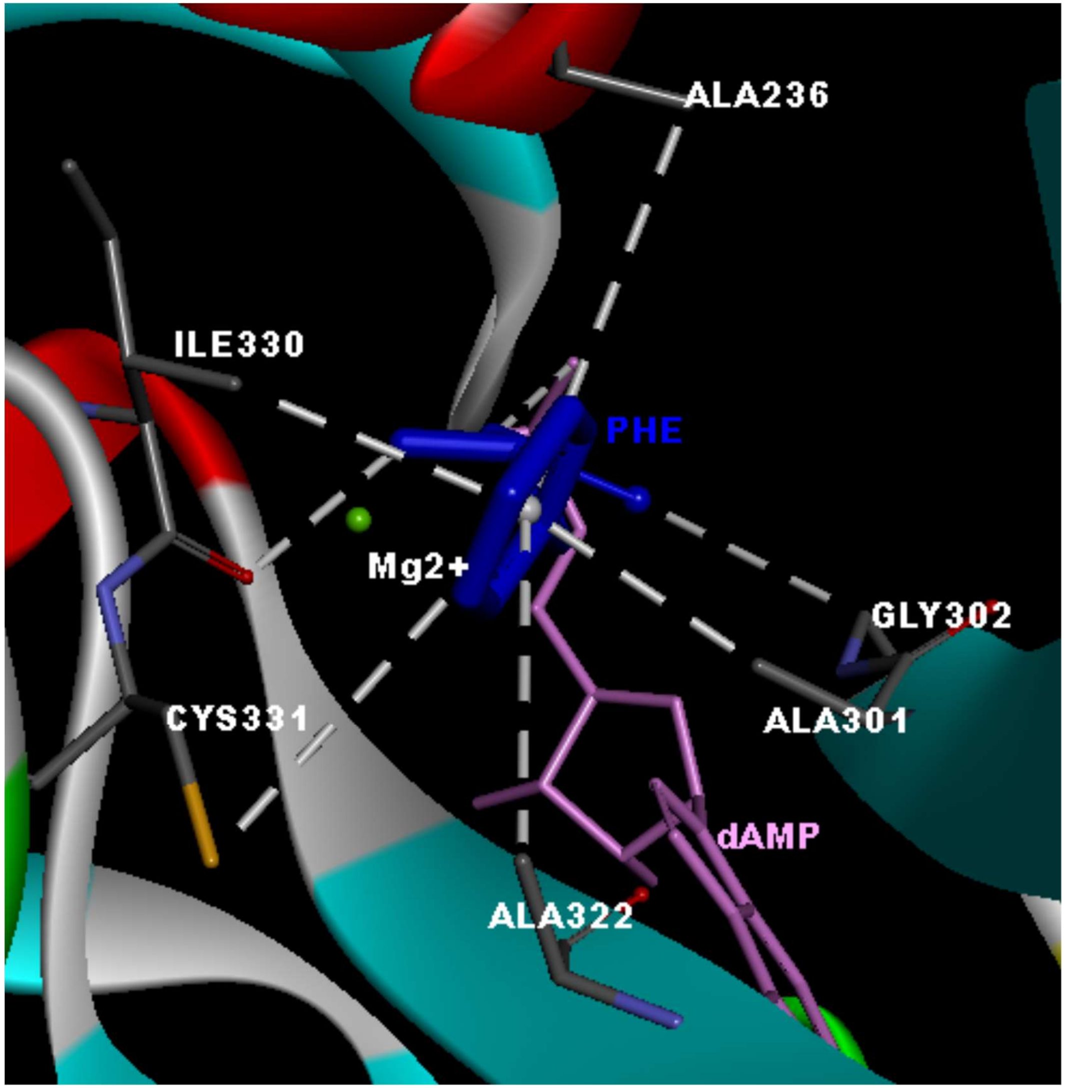

Figure S3

```
.                                                                     I

1 A. migulanus NCTC 7096   100.0%   AGSATSPSLVNKWKEKVTYINAYGPTETTICATTWVATKETIGHSVPIGAPIQNTQIYIVDENLQLKSVGEAGELCIGGEGLARGYWKRPELTSQKFVDN
2 A. migulanus Nagano       99.7%   AGSATSPSLVNKWKEKVTYINAYGPTETTICATTWVATKETTGHSVPIGAPIQNTQIYIVDENLQLKSVGEAGELCIGGEGLARGYWKRPELTSQKFVDN
  consensus/100%                    AGSATSPSLVNKWKEKVTYINAYGPTETTICATTWVATKEThGHSVPIGAPIQNTQIYIVDENLQLKSVGEAGELCIGGEGLARGYWKRPELTSQKFVDN

                                             II
                                401      .          .          .          .          :          .          .          .          .          5 500
1 A. migulanus NCTC 7096   100.0%   PFVPGEKLYKTGDQARWLSDGNIEYLGRIDNQVKIRGHRVELEEVESILLKHMYISETAVSVHKDHQEQPYLCAYFVSEKHIPLEQLRQFSSEELPTYMI
2 A. migulanus Nagano       99.7%   PFVPGEKLYKTGDQARWLPDGNIEYLGRIDNQVKIRGHRVELEEVESILLKHMYISETAVSVHKDHQEQPYLCAYFVSEKHIPLEQLRQFSSEELPTYMI
  consensus/100%                    PFVPGEKLYKTGDQARWLsDGNIEYLGRIDNQVKIRGHRVELEEVESILLKHMYISETAVSVHKDHQEQPYLCAYFVSEKHIPLEQLRQFSSEELPTYMI

                                501      .          .          .          .          :          .          .          .          .          6 600
1 A. migulanus NCTC 7096   100.0%   PSYFIQLDKMPLTSNGKIDRKQLPEPDLTFGMRVDYEAPRNEIEETLVTIWQDVLGIEKIGIKDNFYALGGDSIKAIQVAARLHSYQLKLETKDLLKYPT
2 A. migulanus Nagano       99.7%   PSYFIQLDKMPLTSNGKIDRKQLPEPDLTFGMRVDYEAPRNEIEETLVTIWQDVLGIEKIGIKDNFYALGGDSIKAIQVAARLHSYQLKLETKDLLKYPT
  consensus/100%                    PSYFIQLDKMPLTSNGKIDRKQLPEPDLTFGMRVDYEAPRNEIEETLVTIWQDVLGIEKIGIKDNFYALGGDSIKAIQVAARLHSYQLKLETKDLLKYPT

                                601      .          .          .          .          :          .          .          .          .          7 700
1 A. migulanus NCTC 7096   100.0%   IDQLVHYIKDSKRRSEQGIVEGEIGLTPIQHWFFEQQFTNMHHWNQSYMLYRPNGFDKEILLRVFNKIVEHHDALRMIYKHHNGKIVQINRGLEGTLFDF
2 A. migulanus Nagano       99.7%   IDQLVHYIKDSKRRSEQGIVEGEIGLTPIQHWFFEQQFTNMHHWNQSYMLYRPNGFDKEILLRVFNKIVEHHDALRMIYKHHNGKIVQINRGLEGTLFDF
  consensus/100%                    IDQLVHYIKDSKRRSEQGIVEGEIGLTPIQHWFFEQQFTNMHHWNQSYMLYRPNGFDKEILLRVFNKIVEHHDALRMIYKHHNGKIVQINRGLEGTLFDF
```

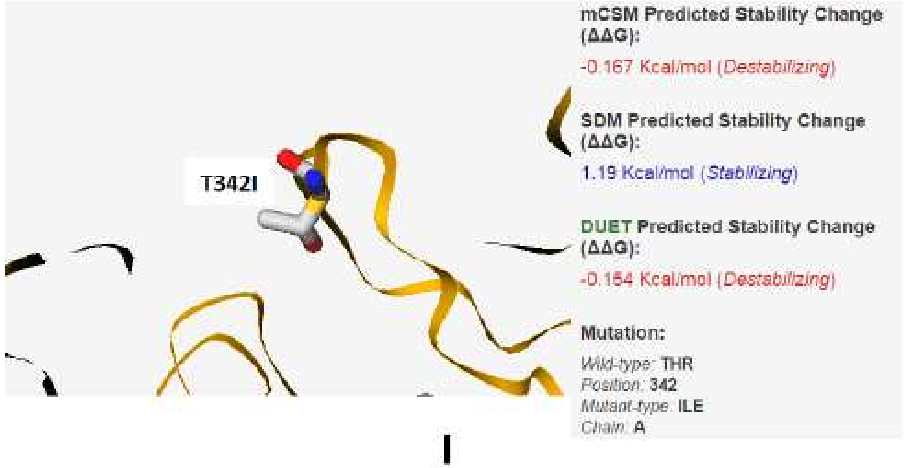

I

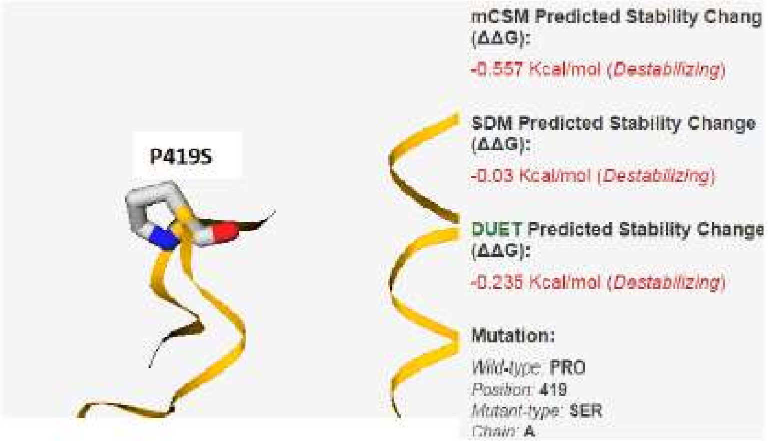

II

Figure S4

(A)

(B)

(C)

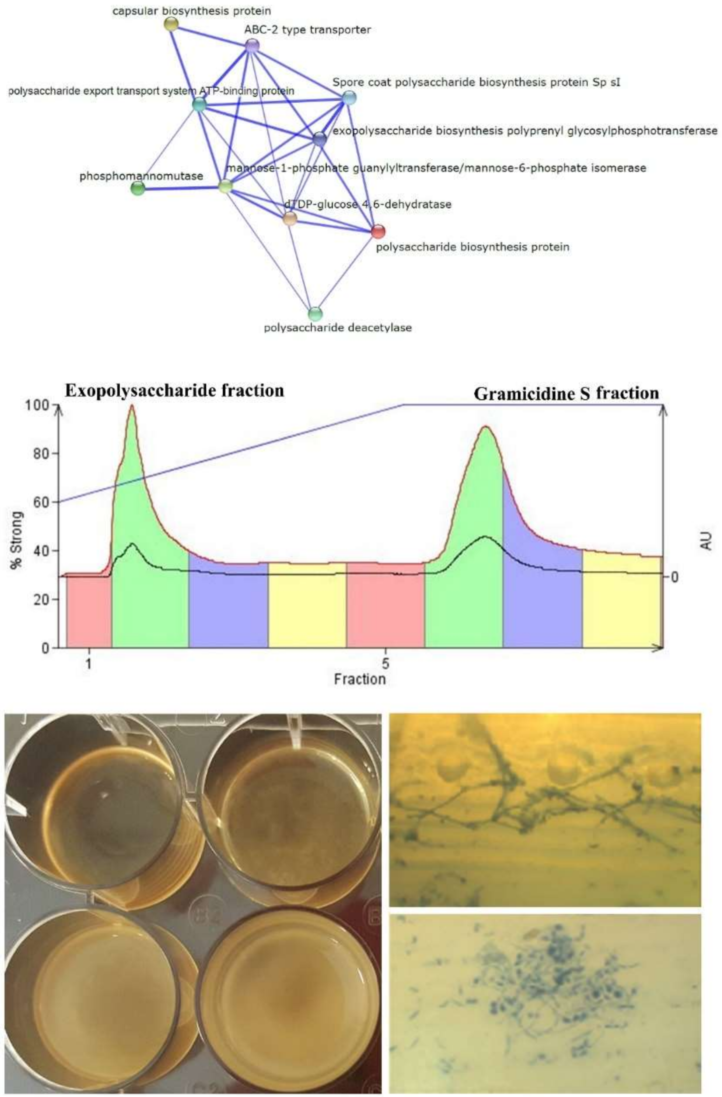

Material and Methods for Figure S4

Biofilm formation by *A. migulanus*

Three mL of TSB were added to the each well in sterilized 12 well cell culture plate (Greiner bio-one, UK), 50 µL of bacterial culture (10^7 bacteria per mL) inoculated and incubated at 37 °C for 3-4 days. Biofilm formation was visualized on the media and around the germinating spores according to Beauregard et al. (2013). Subsequently, samples from the biofilm were harvested and each sample examined under high magnification phase-contrast microscopy.

Protein interaction network of the exopolysaccharide biosynthetic pathway

Ten genes showing strong homology with *Bacillus amyloliquefaciens* genes were identified and envisioned into an interaction network using STRING and GeneMANIA server (Figure 8).

***Dothistroma septosporum* pine infection and pine needle assay**

Pine infection and pine needle assays with *Dothistroma septosporum* were described in Alenezi et al. (2016a).

Table S1

	Nterm_mod	aa1	aa2	aa3	aa4	aa5	aa6	aa7	aa8	aa9	aa10	adduct
	Term	L	F	P	V	K	L	F	P	V	K	[M+H]$^+$
**Mass**	1.007825	113.0841	147.0684	97.05276	99.06841	128.095	113.0841	147.0684	97.05276	99.06841	128.09496	1169.745
b-series	1.007825	114.0919	261.1603	358.2131	457.2815	585.3764	698.4605	845.5289	942.5817	1041.65	1169.745	
Actual mass		-	261.1606	358.2188	457.2803	585.3751	698.4605	845.5264	942.5762	1041.6481		
y-series	1.007825	1169.745	1056.661	909.5926	812.5398	713.4714	585.3764	472.2924	325.224	228.1712	129.102785	
Actual mass		-	1056.6727	909.5892	812.5347	713.4695	585.3751	472.2917	325.2228	228.1687		
	Nterm_mod	aa1	aa2	aa3	aa4	Orn	aa6	aa7	aa8	aa9	aa10	adduct
	Term	L	F	P	V	K	L	F	P	V	K	[M+H]$^+$
**Mass**	1.007825	113.0841	147.0684	97.05276	99.06841	114.0793	113.0841	147.0684	97.05276	99.06841	128.09496	1155.729
b-series	1.007825	114.0919	261.1603	358.2131	457.2815	571.3608	684.4448	831.5132	928.566	1027.634	1155.7294	
Actual mass		-	261.1598	358.2122	457.2795	571.3612	684.4433	831.5117	928.5595	1027.6339		
y-series	1.007825	1155.729	1042.645	895.5769	798.5241	699.4557	585.3764	472.2924	325.224	228.1712	129.102785	
Actual mass			1042.6502	895.5762	798.5214	699.4553	585.3752	472.2917	325.2232	228.1713		
	Nterm_mod	aa1	aa2	aa3	aa4	Orn	aa6	aa7	aa8	aa9	Orn	adduct
	Term	L	F	P	V	K	L	F	P	V	K	[M+H]$^+$
**Mass**	1.007825	113.0841	147.0684	97.05276	99.06841	114.0793	113.0841	147.0684	97.05276	99.06841	113.93846	1141.573
b-series	1.007825	114.0919	261.1603	358.2131	457.2815	571.3608	684.4448	831.5132	928.566	1027.634	1141.5729	
Actual mass			261.1598	358.2108	457.2784	571.3607	684.4442	831.5147	928.5653	1027.6261		
y-series	1.007825	1141.714	1028.63	881.5613	784.5085	685.4401	571.3608	458.2767	311.2083	214.1555	115.087135	
Actual mass			1028.6317	881.5618	784.5082	685.4402	571.3607	458.2810	311.2080	214.1554		

Table S2

	Nterm_mod	aa1	aa2	aa3	aa4	aa5	aa6	aa7	aa8	aa9	aa10	adduct
	Term	L	F	P	V	K	L	F	P	V	K	$[M+H]^+$
**Mass**	1.007825	113.0841	147.0684	97.05276	99.06841	128.095	113.0841	147.0684	97.05276	99.06841	128.09496	1169.745
b-series	1.007825	114.0919	261.1603	358.2131	457.2815	585.3764	698.4605	845.5289	942.5817	1041.65	1169.745	
Actual mass		-	N/A	N/A	N/A	N/A	N/A	N/A	N/A	N/A		
y-series	1.007825	1169.745	1056.661	909.5926	812.5398	713.4714	585.3764	472.2924	325.224	228.1712	129.102785	
Actual mass		-	N/A	N/A	N/A	N/A	N/A	N/A	N/A	N/A		
	Nterm_mod	aa1	aa2	aa3	aa4	Orn	aa6	aa7	aa8	aa9	aa10	adduct
	Term	L	F	P	V	K	L	F	P	V	K	$[M+H]^+$
**Mass**	1.007825	113.0841	147.0684	97.05276	99.06841	114.0793	113.0841	147.0684	97.05276	99.06841	128.09496	1155.729
b-series	1.007825	114.0919	261.1603	358.2131	457.2815	571.3608	684.4448	831.5132	928.566	1027.634	1155.7294	
Actual mass		-	N/A	N/A	457.2799	N/A	684.4435	N/A	N/A	N/A		
y-series	1.007825	1155.729	1042.645	895.5769	798.5241	699.4557	585.3764	472.2924	325.224	228.1712	129.102785	
Actual mass			N/A	895.5762	N/A	N/A	N/A	N/A	325.2242	N/A		
	Nterm_mod	aa1	aa2	aa3	aa4	Orn	aa6	aa7	aa8	aa9	Orn	adduct
	Term	L	F	P	V	K	L	F	P	V	K	$[M+H]^+$
**Mass**	1.007825	113.0841	147.0684	97.05276	99.06841	114.0793	113.0841	147.0684	97.05276	99.06841	113.93846	1141.573
b-series	1.007825	114.0919	261.1603	358.2131	457.2815	571.3608	684.4448	831.5132	928.566	1027.634	1141.5729	
Actual mass			261.1600	358.2123	457.2805	571.3610	684.4446	831.5131	928.5654	1027.6370		
y-series	1.007825	1141.714	1028.63	881.5613	784.5085	685.4401	571.3608	458.2767	311.2083	214.1555	114.946285	
Actual mass			1028.6334	881.5617	784.5094	685.4408	571.3610	458.2766	311.2082	214.1550		

Table S3

GS analogues	Predicted formula	Measured mass $[M+H]^+$	Calculated mass $[M+H]^+$	Error (ppm)
GS-1141	$C_{60}H_{92}N_{12}O_{10}$	1141.7137	1141.7132	0.44
GS-1155	$C_{61}H_{94}N_{12}O_{10}$	1155.7290	1155.7288	0.17
GS-1169	$C_{62}H_{96}N_{12}O_{10}$	1169.7446	1169.7445	0.08

Table S4.

**Proteins in Nagano**	**Proteins in NCTC 7096**
Four 16S ribosomal RNA	Five 16S ribosomal RNA
2-oxoacid:acceptor oxidoreductase, alpha subunit	
	2-oxoglutarate synthase subunit KorA (EC 1.2.7.3)
	2-polyprenyl-3-methyl-5-hydroxy-6-metoxy-1, 4-benzoquinol methylase
	3D-(3,5/4)-trihydroxycyclohexane-1,2-dione hydrolase (THcHDO hydrolase) (EC 3.7.1.22)
Two 4-phosphoerythronate dehydrogenase	
	One 4-phosphoerythronate dehydrogenase
Two 5'-3' exonuclease, SAM domain protein	
	One 5'-3' exonuclease, SAM domain protein
	5-dehydro-2-deoxygluconokinase (EC 2.7.1.92) (2-deoxy-5-keto-D-gluconate kinase)
8-amino-7-oxononanoate synthase 1 (EC 2.3.1.47)	
	AAA ATPase
Two ABC transporter ATP-binding protein	
	Three ABC transporter ATP-binding protein
	Two ABC transporter permease protein
	Three ABC transporter related protein
ABC-2 type transporter	
	ABC transporter, substrate-binding protein, QAT family
Three ABC transporter-like protein	
	Four ABC transporter-like protein
	ABC-type dipeptide/oligopeptide/nickel transport system, ATPase component
	ABC-type multidrug transport system, ATPase component
	ABC-type transport system ATP-binding/permease protein

Proteins in Nagano	Proteins in NCTC 7096
	Abortive infection bacteriophage resistance protein
	Accessory protein regulator protein B
Four Acyltransferase	
	Two Acyltransferase
Acyltransferase 3	
	Acetylornithine aminotransferase (ACOAT) (EC 2.6.1.11)
	Activating signal cointegrator 1
	Adenine specific DNA methylase Mod
Two Adenylyl-sulfate kinase (EC 2.7.1.25) (APS kinase) (ATP adenosine-5'-phosphosulfate 3'-phosphotransferase) (Adenosine-5'-phosphosulfate kinase)	
	Three Adenylyl-sulfate kinase (EC 2.7.1.25) (APS kinase) (ATP adenosine-5'-phosphosulfate 3'-phosphotransferase) (Adenosine-5'-phosphosulfate kinase)
Seven Ala tRNA	
	Four Ala tRNA
Amidase, hydantoinase/carbamoylase family	
	Alanine racemase domain protein
	Aldehyde dehydrogenase (NAD) family protein (EC 1.2.1.-)
	Alkaline phosphatase synthesis transcriptional regulatory protein hop
	Allantoate amidohydrolase
	Three Alpha/beta hydrolase fold protein
Amidase, hydantoinase/carbamoylase family	
	AlwI restriction endonuclease
Four Amino acid carrier protein	
	Five Amino acid carrier protein
Three Amino acid transporter	
	Four Amino acid transporter
One Aminoglycoside phosphotransferase	

Proteins in Nagano	Proteins in NCTC 7096
	Two Aminoglycoside phosphotransferase
Four Aminotransferase class V	
	Three Aminotransferase class V
Three Anti-sigma-factor antagonist	
	Two Anti-sigma-factor antagonist
One Arabinose efflux permease family protein	
	Three Arabinose efflux permease family protein
Two AraC family transcriptional regulator	
	Three AraC family transcriptional regulator
AraC-like ligand binding domain protein	
One Arginase	
	Two Arginase
Five Asn tRNA	
	Four Asn tRNA
Eight Asp tRNA	
	Eleven Asp tRNA
	Asparagine synthase
One ATPase	
	Two ATPase
Two ATPase involved in DNA repair	
	ATPase AAA
ATP-binding protein	
ATP-dependent Clp protease proteolytic subunit	
	Five ATPase/histidine kinase/DNA gyrase B/HSP90 domain protein (Fragment)
ATP-dependent DNA helicase UvrD	
Three ATP-dependent zinc metalloprotease FtsH (EC 3.4.24.-)	One ATP-dependent zinc metalloprotease FtsH (EC 3.4.24.-)

**Proteins in Nagano**	**Proteins in NCTC 7096**
One Bacitracin transport ATP-binding protein bcrA	
	Two Bacitracin transport ATP-binding protein bcrA
One Bacitracin transport permease protein BcrC	
	Three Bacitracin transport permease protein BcrC
Two Bacterial ABC transporter protein EcsB	
	One Bacterial ABC transporter protein EcsB
Two Bacterial RNA polymerase, alpha chain domain protein	
Two Bacterial transferase hexapeptide repeat protein (EC 2.3.1.28)	
One Baseplate J family protein	
	Two Baseplate J family protein
	Two Baseplate J-like protein
Twelve Beta-lactamase	
	Nine Beta-lactamase
ChiA12	
	Beta-lactamase domain protein
Two CAAX amino terminal protease family protein	
	Three CAAX amino terminal protease family protein
	Capsular exopolysaccharide family protein
	Cell division membrane protein
	Two Chain length determinant protein
Conjugation protein, TraG/TraD family	
	Cupin
Two Cupin domain protein	
	Three Cupin domain protein

**Proteins in Nagano**	**Proteins in NCTC 7096**
Three Cyclic pyranopterin monophosphate synthase (EC 4.1.99.18) (Molybdenum cofactor biosynthesis protein A)	
	Two Cyclic pyranopterin monophosphate synthase (EC 4.1.99.18) (Molybdenum cofactor biosynthesis protein A)
One Cysteine-rich domain protein (Fragment)	
	Three Cysteine-rich domain protein (Fragment)
	Cytochrome aa3 quinol oxidase, subunit I
DegT/DnrJ/EryC1/StrS aminotransferase	
Two Dehydrogenase	
	One Dehydrogenase
	Diaminopimelate epimerase
Seven Diguanylate cyclase domain protein	
	Eight Diguanylate cyclase domain protein
	Dihydrodipicolinate synthase/N-acetylneuraminate lyase
One DinB family protein	
	Two DinB family protein
One D-isomer specific 2-hydroxyacid dehydrogenase NAD-binding	
	Two D-isomer specific 2-hydroxyacid dehydrogenase NAD-binding
	DNA integrase
	DNA methyltransferase
DNA N-6-adenine-methyltransferase	
DNA polymerase III subunit beta (EC 2.7.7.7)	
	DNA polymerase III subunit epsilon
DNA polymerase III, epsilon subunit (EC 2.7.7.7)	
	DNA segregation ATPase FtsK/SpoIIIE

**Proteins in Nagano**	**Proteins in NCTC 7096**
DNA polymerase V (EC 2.7.7.7)	
DNA primase (EC 2.7.7.-)	
One DNA topoisomerase (EC 5.99.1.2)	
	Two DNA topoisomerase (EC 5.99.1.2)
DNA sulfur modification protein DndB	
One DNA-3-methyladenine glycosylase (EC 3.2.2.21)	
	Two DNA-3-methyladenine glycosylase (EC 3.2.2.21)
One DnaJ domain protein	
	Two DnaJ domain protein
	DnaJ-class molecular chaperone with C-terminal Zn finger domain
	Three Domain protein
dTDP-glucose 4,6-dehydratase (EC 4.2.1.46)	
	D-ribose transporter subunit RbsB
ECF subfamily RNA polymerase sigma-24 subunit	
	DUTPase
	Efflux transporter, RND family, MFP subunit family protein
Three Electron transfer flavoprotein subunit alpha	
	Four Electron transfer flavoprotein subunit alpha
Three Electron transfer flavoprotein subunit beta	
	One Electron transfer flavoprotein subunit beta
	EmrB/QacA subfamily drug resistance transporter
Two Esterase	
	One Esterase
Two Erythromycin esterase	
	Three Erythromycin esterase

**Proteins in Nagano**	**Proteins in NCTC 7096**
Exopolysaccharide biosynthesis polyprenyl glycosylphosphotransferase	
One FHA domain protein	
	Two FHA domain protein
Two Flagellar hook-basal body protein	
	Three Flagellar hook-basal body protein
Flagellar protein FliS	
	FOG: transposase and inactivated derivative (Fragment)
	Fosmidomycin resistance protein
One Fructose-1,6-bisphosphate aldolase, class II	
	Two Fructose-1,6-bisphosphate aldolase, class II
	G/T mismatches repair enzyme (EC 3.2.2.-)
	GalE
	Gallidermin-class lantibiotic protection ABC transporter, ATP-binding subunit
Twelve Glu tRNA	Nine Glu tRNA
	Glucose 1-dehydrogenase
	Glucose uptake protein GlcU
One Glucose-6-phosphate 1-dehydrogenase (G6PD) (EC 1.1.1.49)	
	Two Glucose-6-phosphate 1-dehydrogenase (G6PD) (EC 1.1.1.49)
	Glutamate synthase family protein
Two Glutaminase (EC 3.5.1.2)	
	Three Glutaminase (EC 3.5.1.2)
	Glutamine permease, sodium/alanine symporter family protein

**Proteins in Nagano**	**Proteins in NCTC 7096**
Two Glutathione peroxidase	
	Three Glutathione peroxidase
One Glycine/D-amino acid oxidase, deaminating	
	Two Glycine/D-amino acid oxidase, deaminating
One Glycosyl hydrolase family 25	
	Two Glycosyl hydrolase family 25
Two Glycosyl transferase group 1	
	One Glycosyl transferase group 1
Glycosyltransferase similar 95% with Aneurinibacillus aneurinilyticus (first one in the excel sheet)	
Four Glycosyltransferase, group 2 family protein	
	Six Glycosyltransferase, group 2 family protein
Seven Glycosyltransferase, group 1 family protein	
	Six Glycosyltransferase, group 1 family protein
Gp66	
	Glycosyltransferase, group 2 family protein (EC 2.4.-.-)
Group II intron-encoded protein ltrA	
	Gp128
Three HNH endonuclease domain protein	
	One HNH endonuclease domain protein
Three Holliday junction resolvase RecU (EC 3.1.22.-) (Recombination protein U homolog)	

**Proteins in Nagano**	**Proteins in NCTC 7096**
	One Holliday junction resolvase RecU (EC 3.1.22.-) (Recombination protein U homolog)
	HPr kinase
One ICEBs1 integrase	
	Two ICEBs1 integrase
Inosose dehydratase (EC 4.2.1.44) (2-keto-myo-inositol dehydratase)	
Two Integral membrane protein domain protein	
	One Integral membrane protein domain protein
Four Insertion element protein	
	Five Insertion element protein
One Integral membrane sensor signal transduction histidine kinase	
	Four Integral membrane sensor signal transduction histidine kinase
	Integral membrane sensor hybrid histidine kinase
	Integrase catalytic region
	Four Integrase catalytic subunit
	Integrase core domain protein (Fragment)
	Iron ABC transporter substrate-binding protein
	Iron dicitrate transporter
One IS1236 transposase domain protein (Fragment)	
	Two IS1236 transposase domain protein (Fragment)
	IS1236 transposase family protein
Isoleucyl-tRNA synthetase	
	Isochorismatase
	Isoleucine--tRNA ligase 2 (EC 6.1.1.5)
One IstB-like ATP-binding protein	
	Two IstB-like ATP-binding protein

**Proteins in Nagano**	**Proteins in NCTC 7096**
	Kelch repeat protein
	Lantibiotic protection ABC transporter permease subunit, MutE/EpiE family
	Lantibiotic protection ABC transporter permease subunit, MutG family
Two LexA DNA binding domain protein	
	One LexA DNA binding domain protein
Three LexA repressor (EC 3.4.21.88)	
	One LexA repressor (EC 3.4.21.88)
One LPXTG-motif protein cell wall anchor domain protein	
	Two LPXTG-motif protein cell wall anchor domain protein
Two Lys tRNA	LysR family transcriptional regulator
	Major facilitator superfamily protein
Two Mannosyl-glycoprotein endo-beta-N-acetylglucosaminidase	
	One Mannosyl-glycoprotein endo-beta-N-acetylglucosaminidase
Five MarR family transcriptional regulator	
	Four MarR family transcriptional regulator
Four Membrane protein	
	Five Membrane protein
Membrane-bound lytic murein transglycosylase F	
	Membrane-associated sensory histidine kinase
Nine Met tRNA	
	Twelve Met tRNA
One Methylmalonate semialdehyde dehydrogenase [acylating] (MMSA dehydrogenase) (MMSDH) (MSDH) (EC 1.2.1.27) (Malonate semialdehyde dehydrogenase [acetylating])	

**Proteins in Nagano**	**Proteins in NCTC 7096**
	Two Methylmalonate semialdehyde dehydrogenase [acylating] (MMSA dehydrogenase) (MMSDH) (MSDH) (EC 1.2.1.27) (Malonate semialdehyde dehydrogenase [acetylating])
Methylmalonyl-CoA epimerase	
	Methylated-DNA--protein-cysteine methyltransferase (EC 2.1.1.63) (6-O-methylguanine-DNA methyltransferase) (O-6-methylguanine-DNA-alkyltransferase)
One Methylphosphotriester-DNA alkyltransferase	
	Two Methylphosphotriester-DNA alkyltransferase
	Methyltransferase
	Two Methyltransferase type 11
Two Methyltransferase type 12	
	Three Methyltransferase type 12
	MFS transporter
	MFS-type transporter ydeR
	Modification methylase BbvI (M.BbvI) (EC 2.1.1.37) (Cytosine-specific methyltransferase BbvI)
	Molecular chaperone, HSP90 family protein
One MOSC domain protein	
	Two MOSC domain protein
	Two Multidrug ABC transporter ATPase
	Multidrug-efflux transporter
	Na(+) extrusion ABC transporter ATP-binding protein
Six N-acetylmuramoyl-L-alanine amidase	
	Fourteen N-acetylmuramoyl-L-alanine amidase
Two N-acetyltransferase GCN5	
	One N-acetyltransferase GCN5
NDP-sugar epimerase, involve in polysaccharide synthesis	
	NADH dehydrogenase subunit 5

**Proteins in Nagano**	**Proteins in NCTC 7096**
	Nickel ABC transporter periplasmic nickel-binding protein
Nickel transport system (Permease)	
	Nickel transporter permease nikB
	Nickel-binding periplasmic protein
Nitrate/nitrite transporter	
	N-terminal phage replisome organizer
Two Nitroreductase	
	Three Nitroreductase
Two Nuclease-like protein	
	One Nuclease-like protein
PBSX phage terminase small subunit	
	Oxidoreductase, NAD-binding domain protein
	PadR family transcriptional regulator
Two Penicillin-binding protein	
	Three Penicillin-binding protein
	Pentapeptide repeat protein
One Peptidase C45 acyl-coenzyme A:6-aminopenicillanic acid acyl-transferase	
	Two Peptidase C45 acyl-coenzyme A:6-aminopenicillanic acid acyl-transferase
	Two Peptidase M15
	Two Peptidase, M56 family
One Peptide ABC transporter ATP-binding protein	
	Two Peptide ABC transporter ATP-binding protein
One Periplasmic copper-binding protein	
	Two Periplasmic copper-binding protein
	Periplasmic component of abc-type transport system
	Phage integrase family protein

**Proteins in Nagano**	**Proteins in NCTC 7096**
	Two Phage portal protein, SPP1 family
	Three Phage protein
Phage protein Gp37/Gp68	
Two Phage integrase, SAM-like domain protein	
	One Phage integrase, SAM-like domain protein
Phage major tail protein, phi13 family	
	Phage portal protein
Phage minor structural protein	
	Phage recombination protein Bet
Phage portal protein	
	Phage tail tape measure protein, TP901 family, core region
Phage tail component protein	
	Phage terminase, large subunit, PBSX family
	Phage-like element PBSX protein xkdF
	Phage-like element PBSX protein XkdP
Two Phage XkdN-like protein	
	One Phage XkdN-like protein
Phosphoribosylformylglycinamidine synthase, purS protein	
One Phosphoserine aminotransferase (EC 2.6.1.52) (Phosphohydroxythreonine aminotransferase)	
	Two Phosphoserine aminotransferase (EC 2.6.1.52) (Phosphohydroxythreonine aminotransferase)
One Pirin family protein	
	Three Pirin family protein
	Platelet-activating factor acetylhydrolase, plasma/intracellular isoform II
One Pleiotropic regulatory protein DegT	

**Proteins in Nagano**	**Proteins in NCTC 7096**
	Three Pleiotropic regulatory protein DegT
	Polysaccharide biosynthesis protein CapD
Eight Polysaccharide deacetylase	
	Ten Polysaccharide deacetylase
Protein translocase subunit SecE	
	Putative capsular polysaccharide biosynthesis protein
Protein virB4	
	Putative aspartate transaminase
Putative amino acid efflux protein	
	Putative acetyltransferase
Putative DNA-invertase hin	
	Two Proline racemase
One Protein involved in initiation of plasmid replication	
	Two Protein involved in initiation of plasmid replication
Putative nuclease (EC 3.1.31.1)	
	Protein MurJ homolog
Putative nucleoside-diphosphate sugar epimerase	
	Protein phosphatase 2C
Putative phage head-tail adaptor	
	Two Protein-export membrane protein, SecD/SecF family
Putative phage terminase, large subunit	
	Pseudaminic acid biosynthesis-associated protein PseG
Putative polysaccharide export transport system ATP-binding protein	
	Putative choline ABC transporter, ATP-binding protein OpuBA

**Proteins in Nagano**	**Proteins in NCTC 7096**
Putative prophage LambdaBa02, tape measure protein	
	Six Putative delta-1-pyrroline-5-carboxylate dehydrogenase
One Putative membrane-bound metalloprotease	
	Two Putative membrane-bound metalloprotease
Putative phage anti-repressor	
	Putative phage major head subunit gpT
	Putative plasmid-partitioning protein
Two Putative response regulator protein GraR	
	Three Putative response regulator protein GraR
	Putative S-layer protein
	Four Putative spore germination protein
	Putative tail tape measure protein
	Putative threonine-rich GPI-anchored glycoprotein (Fragment)
One Putative transposase	
	Three Putative transposase
	Putative transposase for insertion sequence element IS3 family
	Putative transposase InsK for insertion sequence element IS150

**Proteins in Nagano**	**Proteins in NCTC 7096**
	Two Putative transposase y4qE
One Recombination and repair protein RecT	
	Three Recombination and repair protein RecT
Two Replicative DNA helicase	
	Six Replicative DNA helicase
Putative type I restriction-modification system methyltransferase subunit	
	Repressor LexA
Response regulator RpfG	
	Response regulator containing a CheY-like receiver domain and an HTH DNA-binding domain
Response regulator containing CheY-like receiver domain and AraC-type DNA-binding domain	
Two Putative toxin-antitoxin system, toxin component	
	One Putative toxin-antitoxin system, toxin component
Three Rad52/22 double-strand break repair protein	
	Two Rad52/22 double-strand break repair protein
Four RHS repeat-associated core domain-containing protein	
Ribosomal RNA large subunit methyltransferase J	
RNA polymerase sigma factorAlprotein, QAT family	
RNA polymerase sigma-70 factor, ECF subfamily (EC 2.7.7.6)	
RNA polymerase sigma-K factor	
Three RNA polymerase, sigma-24 subunit, ECF subfamily	
	Two RNA polymerase, sigma-24 subunit, ECF subfamily

**Proteins in Nagano**	**Proteins in NCTC 7096**
Nine RND transporter, HAE1/HME family, permease protein	
	Four RND transporter, HAE1/HME family, permease protein
SacI restriction endonuclease	
	Rrf2 family protein
Two S4 domain protein	
	Four S4 domain protein
	S23 ribosomal protein
One S-adenosyl-L-methionine-dependent methyltransferase	
	Two S-adenosyl-L-methionine-dependent methyltransferase
Sigma-70 family RNA polymerase sigma factor	
	Scaffold protein
Nine Sigma-70 region 2	
	Ten Sigma-70 region 2
Three Signal peptidase I (EC 3.4.21.89)	
	Two Signal peptidase I (EC 3.4.21.89)
Site-specific DNA methylase	
	Site-specific recombinase, phage integrase family
Site-specific integrase	
Three Site-specific recombinase XerD	
Two S-layer homology domain protein	
	One S-layer homology domain protein
Small subunit ribosomal protein S1 (EC 1.17.1.2)	
One SMI1 / KNR4 family protein	
	Two SMI1 / KNR4 family protein
One Sortase family protein	

**Proteins in Nagano**	**Proteins in NCTC 7096**
	Two Sortase family protein
Spore coat polysaccharide biosynthesis protein SpsI	
	Two Spore germination protein (Amino acid permease)
Two Spore coat protein, CotS family	
	One Spore coat protein, CotS family
Succinate-semialdehyde dehydrogenase (NADP+) GabD (EC 1.2.1.79)	
	Succinate-semialdehyde dehdyrogenase (EC 1.2.1.16)
	Sugar transferase
Two Sugar O-acyltransferase, sialic acid O-acetyltransferase NeuD family	
	One Sugar O-acyltransferase, sialic acid O-acetyltransferase NeuD family
Sulfurtransferase DndC	
Two Surface-layer glycoprotein SatB	
Terminase-like family	
	Terminase large subunit
Two Thioredoxin reductase	
Three Thermonuclease family protein	
	Five Thermonuclease family protein
Two Thioredoxin	
	Three Thioredoxin
One Threonine synthase	
	Two Threonine synthase
TIGR02436 family protein	
	Tn7-like transposition protein C
TnsA endonuclease	
	Tn7-like transposition protein D
	TnsA endonuclease domain protein

**Proteins in Nagano**	**Proteins in NCTC 7096**
	Topoisomerase DNA-binding C4 zinc finger domain protein
	Toxin-antitoxin system, antitoxin component, HicB family
	Transcriptional regulator for pyruvate dehydrogenase complex, GntR family protein
Three Transcriptional regulator, AsnC family	
	Four Transcriptional regulator, AsnC family
One Transcriptional regulator, BlaI/MecI/CopY family	
	Three Transcriptional regulator, BlaI/MecI/CopY family
	Transcriptional regulator, DeoR family
One Translocator protein, LysE family	
	Two Translocator protein, LysE family
Three Transporter	
	Four Transporter
Two Transposase (15)	
Transposase for transposon Tn1546	
	One Transposase (15)
Transposase domain (DUF772)	
Transposase IS200/IS605 family	
	Transposase for insertion sequence element IS3 family protein
One Transposase family protein	
	Three Transposase family protein
	Transposase IS116/IS110/IS902 family protein
Transposase TnpA, ISL3 family	
	Transposase IS3/IS911 family protein
Two Transposase IS4 family protein	
	Five Transposase IS4 family protein
Transposase IS66 family (Fragment)	
	Two Transposase IS66

**Proteins in Nagano**	**Proteins in NCTC 7096**
Transposase TnpA, ISL3 family	
	Transposase IS4 family protein (Fragment)
	Transposase of IS5377-like element
	Transposase, IS4 family (Fragment)
	Transposase, IS605 OrfB family
Nine Transposase, IS4 family	
	Seven Transposase, IS4 family
One Transposase, IS605 OrfB family, central region	
	Three Transposase, IS605 OrfB family, central region
	Transposase-like protein
	Triple helix repeat-containing collagen
	Tryptophan--tRNA ligase
	Six of Two-component hybrid sensor and regulator
	Two-component protein kinase (EC 2.7.3.-)
Type II secretion system protein E	
	Type III restriction enzyme, res subunit
Five Tyr tRNA	
	Four Tyr tRNA
One Tyrosine recombinase XerC	
	Two Tyrosine recombinase XerC
Tyrosine recombinase xerS	
UDP-galactopyranose mutase	
	UDP-N-acetylglucosamine 2-epimerase (EC 5.1.3.14) (Fragment)
Uncharacterized conserved protein	
	Uncharacterized oxidoreductase CzcO-like (EC 1.-.-.-)
Uncharacterized protein	
	1199 Uncharacterized protein

**Proteins in Nagano**	**Proteins in NCTC 7096**
14 Uncharacterized protein (Fragment)	
	14 Uncharacterized protein (Fragment)
Uncharacterized proteinII	
	Uncharacterized protein YcgI
	Uncharacterized protein yqjZ
Uridine kinase (EC 2.7.1.48)	
	UPF0061 protein HMPREF0083_01523
	UPF0753 protein Theco_3474
Three UTP-glucose-1-phosphate uridylyltransferase	
	Two UTP-glucose-1-phosphate uridylyltransferase
Eight Val tRNA	
	Six Val tRNA
One YqaJ viral recombinase family protein	
	Two YqaJ viral recombinase family protein
YoqW	
	YvbHGln tRNA
YvbH	
